# BRAIN-TIME: Testing Whether Prospective Recovery Position Modifies the Benefit of the Same Intervention in Addiction

**DOI:** 10.3390/brainsci16101043

**Published:** 2026-09-29

**Authors:** Anna Makarewicz, Remigiusz Recław, Anna Grzywacz, Krzysztof Chmielowiec, Łukasz Jaworski, Marta Kuczak-Wójtowicz, Jolanta Chmielowiec

**Affiliations:** 1 Department of Hygiene and Epidemiology, Collegium Medicum, University of Zielona Góra, 28 Zyty St., 65-046 Zielona Góra, Poland; makarewicz81@gmail.com (A.M.); chmiele@vp.pl (K.C.); 2 Department of Public Health, Epidemiology and Behavioral Genetics, Pomeranian Medical University in Szczecin, Powstancow Wielkopolskich 72 St., 70-111 Szczecin, Poland; remigiusz.reclaw@pum.edu.pl; 3 Department of Molecular Biology, Gdansk University of Physical Education and Sport, Kazimierza Gorskiego 1 St., 80-336 Gdansk, Poland; 4 Cardiac Surgery Department, Specialized Hospital in Wejherowo, Jagalskiego 10 St., 84-200 Wejherowo, Poland; ljaworski75@gmail.com; 5 Independent Public Specialist Health Care Facility “ZDROJE”, Mączna 4 St., 70-780 Szczecin, Poland; martakuczak@wp.pl; 6 Department of Nursing, Collegium Medicum, University of Zielona Góra, 28 Zyty St., 65-046 Zielona Góra, Poland; j.chmielowiec@inz.uz.zgora.pl

**Keywords:** addiction neuroscience, substance use disorders, craving, cue reactivity, recovery dynamics, temporal actionability, dynamic prediction, treatment-effect heterogeneity, treatment timing, BRAIN-TIME

## Abstract

**Highlights:**

**What are the main findings?**

- This Perspective proposes, without empirical testing, an explicit evidentiary qualification layer between prospective recovery information and treatment-timing policy: prospective episode position is argued to become decision-relevant only if it modifies the benefit of the same intervention.
- BRAIN-TIME is structured to separate perturbation-anchored trajectory measurement, retrospective recovery adjudication, prospective estimation restricted to information available at the decision point, and randomized treatment-effect moderation, so that prediction and temporal actionability can be distinguished empirically in future studies.

**What are the implications of the main findings?**

- If the proposal is correct, mechanistic plausibility and predictive accuracy should not be interpreted as evidence of temporal actionability, which would instead require prospective demonstration of intervention-specific effect modification.
- The framework specifies in advance what would count as support and what would count as refutation: a positive Stage 1 result would justify, but not replace, a subsequent timing-policy trial, whereas an adequately informative null or practically negligible treatment-by-recovery-position effect would falsify temporal actionability for the tested intervention and context. No such test has yet been conducted.

**Abstract:**

Addiction neuroscience has advanced the identification of vulnerability and mechanisms involving reward learning, salience, cognitive control, interoception, cue reactivity, and stress regulation. Yet a key translational question remains unresolved: when does an evolving post-perturbation brain–behavior trajectory become relevant to treatment benefit? Prediction models can estimate future craving, lapse, or deterioration, and adaptive-intervention systems can deliver support using prespecified tailoring variables, but neither dynamic treatment regimes, just-in-time adaptive interventions, nor causal machine learning specifies what evidence a temporal quantity must satisfy before it may serve as the tailoring variable itself. This Perspective asks whether prospective recovery position modifies the benefit of the same intervention, and introduces BRAIN-TIME as a falsifiable episode-level architecture for answering that question. It begins with a defined cue, stress, affective, or sleep-related perturbation, follows one prespecified recovery domain relative to an individualized reference, and separates retrospectively adjudicated sustained re-entry from a prospective estimate of incomplete recovery computed only from information available at the decision point, with no use of later observations. Its decisive Stage 1 test is treatment-effect moderation: at a common locked landmark, the benefit of the same intervention and dose must vary across prospectively estimated recovery positions by a prespecified, practically meaningful amount. A minimal proof of concept combines a standardized low-intensity challenge, repeated craving or autonomic sampling, an independent outward-function criterion, negative controls, and randomized fixed-content intervention. No original data are reported, and every quantity specified here is a proposed construct, estimand, or falsification criterion rather than an observed result. BRAIN-TIME is therefore neither a relapse classifier nor an autonomous treatment rule. Its central claim is deliberately narrow: temporal recovery information becomes relevant to treatment timing only when prospective episode position is shown to modify the benefit of the same intervention.

## 1. Introduction

Addiction research increasingly addresses two foundational questions: who is vulnerable and why addictive behavior becomes persistent. Person-level frameworks integrate regulatory control, clinical context, and biological or behavioral indicators, whereas mechanistic frameworks focus on reward learning, cue reactivity, salience, cognitive control, interoception, and stress regulation. A third question remains comparatively underdeveloped: when does an evolving post-perturbation trajectory become relevant to treatment benefit? Within BRAIN-TIME, risk and mechanistic findings are treated as upstream evidence; they become relevant to treatment timing only when prospectively available episode information is shown to modify intervention benefit.

The same observed symptom severity may represent very different positions within an episode. A craving score of six, for example, may occur during rapid resolution, plateau, rebound, or prolonged deviation. Throughout this article, recovery refers specifically to episode-level restoration of one prespecified domain following a defined perturbation, not to long-term recovery from addiction, remission, or recovery capital. Neither symptom severity nor elapsed time necessarily captures trajectory position: subjective duration judgments are influenced by interoceptive, sensorimotor, and emotional processes [1]. Contemporary psychiatry increasingly conceptualizes mental disorders as dynamical systems characterized by persistence, transitions, context dependence, and reconfiguration [2,3], consistent with dynamical-systems approaches to precision psychiatry [4] and broader biological accounts of resilience and robustness as temporally dynamic responses to perturbation [5]. These perspectives motivate the study of recovery dynamics but do not establish when temporal information should alter treatment decisions.

Mechanistic evidence further supports the plausibility of temporally organized recovery. Allostasis has been proposed as a fundamental organizing principle of brain function [6], and ultrahigh-field imaging has identified distributed cortical and subcortical allostatic–interoceptive systems involved in regulating the internal milieu [7]. Large-scale cortical activity exhibits structured cycles [8], while focal thalamic input can trigger rapid brain-wide network reconfiguration [9]. Neural dynamics are experimentally modifiable through state-dependent stimulation [10], shaped by cell-type-specific plasticity during action-timing learning [11], distributed across multiple neural timescales [12], and embedded within hierarchical brain–body rhythms [13]. Together, these findings support the view that brain function is dynamic, multiscale, and temporally organized. They do not, however, identify a treatment window.

Prediction alone does not resolve this problem. A model may accurately forecast craving persistence, lapse, or physiological dysregulation without identifying a position at which treatment becomes more or less beneficial. Early-warning research illustrates this distinction: statistical precursors of change do not automatically constitute validated intervention triggers [14]. BRAIN-TIME therefore separates prospective informativeness from temporal actionability. An episode may be measurable and prospectively estimable without demonstrating that intervention benefit varies with its estimated position.

Four terms recur throughout this article and are defined here in plain language. An episode is the period that begins with a defined perturbation, such as a brief cue or stress exposure, and ends when the affected measure has returned to the person’s own usual range and remained there, or when observation stops. A decision landmark is a fixed moment within that episode, chosen in advance, at which a treatment decision could be made. Prospective recovery position is an estimate, made at that moment and using only information already available, of how likely it is that recovery will still be incomplete some time later; it describes where the person stands within the episode, not a diagnosis or a brain state. Temporal actionability means that this position changes how much the same intervention helps: a position is actionable only if the intervention works measurably better at some positions than at others.

Recent state-dependent, closed-loop, and trajectory-based studies provide important evidence that neural state and temporal context can influence intervention effects. However, they have not yet tested the perturbation-anchored, prospectively estimable recovery construct developed here. The following sections position BRAIN-TIME in relation to these adjacent studies and specify the evidentiary sequence required to evaluate its central claim.

## 2. Conceptual Scope and Evidentiary Logic

This Perspective develops a hypothesis-driven validation framework grounded in a critical synthesis of relevant evidence. It does not estimate the prevalence of prolonged recovery, identify a universal intervention window, or claim that decision timing is intrinsically encoded in every physiological or neural signal. The reviewed evidence is used to construct and constrain a testable sequence linking perturbation, trajectory, prospective inference, and treatment effect.

The primary evidence domains are dynamical neuroscience, neural timescales, allostatic and interoceptive regulation, cue–craving–use dynamics, intensive longitudinal addiction research, dynamic prediction, prediction-model evaluation, and time-varying treatment-effect estimation. Representative sources for each domain are cited at the point where that domain’s evidence is used (Section 1, Section 4, and Section 6). Sources were identified through PubMed/MEDLINE and publisher databases, supplemented by citation tracking from retrieved articles, with searches conducted through mid-2026. Recent sources from 2024 onward were prioritized where they provided updated empirical evidence on measurement, modelling, addiction mechanisms, or intervention effects; primary studies and randomized designs were preferentially used to support specific empirical claims, whereas high-quality reviews and perspectives were retained where they provided necessary conceptual or methodological synthesis. Established sources predating this period were retained when methodological or inferential precision required them.

The synthesis also deliberately retained disconfirming or null evidence where available rather than selecting only findings consistent with the WHEN hypothesis. The high-intensity exercise trial reviewed in Section 5.3 and the early-warning critique [14] are included for this reason. The literature synthesis was intended to identify and constrain the evidentiary foundations of the framework rather than to provide an exhaustive systematic review. Sources were selected for their relevance to the specific mechanistic, predictive, and causal claims developed here. Accordingly, the evidence supporting several claims in Section 6 remains a small set of individually informative recent studies rather than a mature, convergent body of evidence; this limitation is stated explicitly and revisited in Section 12.

The evidentiary sequence is cumulative. Measurement credibility precedes trajectory interpretation; trajectory interpretation precedes prospective estimation; prospective estimation precedes a treatment-timing interaction; and a treatment-timing interaction precedes any claim of clinical utility. Failure at an earlier stage cannot be rescued by a later association. This ordering prevents evidentiary strength from being inferred backwards across levels: each claim must be supported at its own inferential stage rather than borrowing plausibility from a later, stronger-sounding result.

## 3. Conceptual Separation of Vulnerability, Mechanism, and Treatment Timing

Conceptual continuity across a research programme is valuable only if successive frameworks address distinct scientific questions, operate at different units of analysis, and require different decisive tests. BRAIN-TIME is therefore positioned explicitly relative to two earlier frameworks rather than presented as a relabelled extension. Table 1 summarizes these distinctions.

MAC/MAB–RCS addresses person-level regulatory stratification, whereas Reward Instability Theory addresses the dynamical organization of reward processing. BRAIN-TIME does not introduce a second latent regulatory state or a second theory of reward instability. Instead, it asks whether a defined episode contains a prospectively detectable position at which the benefit of the same intervention changes. This distinction is structural rather than rhetorical: the WHO and WHY frameworks may be valid even if the WHEN hypothesis is ultimately falsified.

The same conceptual discipline applies when BRAIN-TIME is compared with established prediction and intervention-timing paradigms outside this research programme (Table 2).

**Figure 1:**
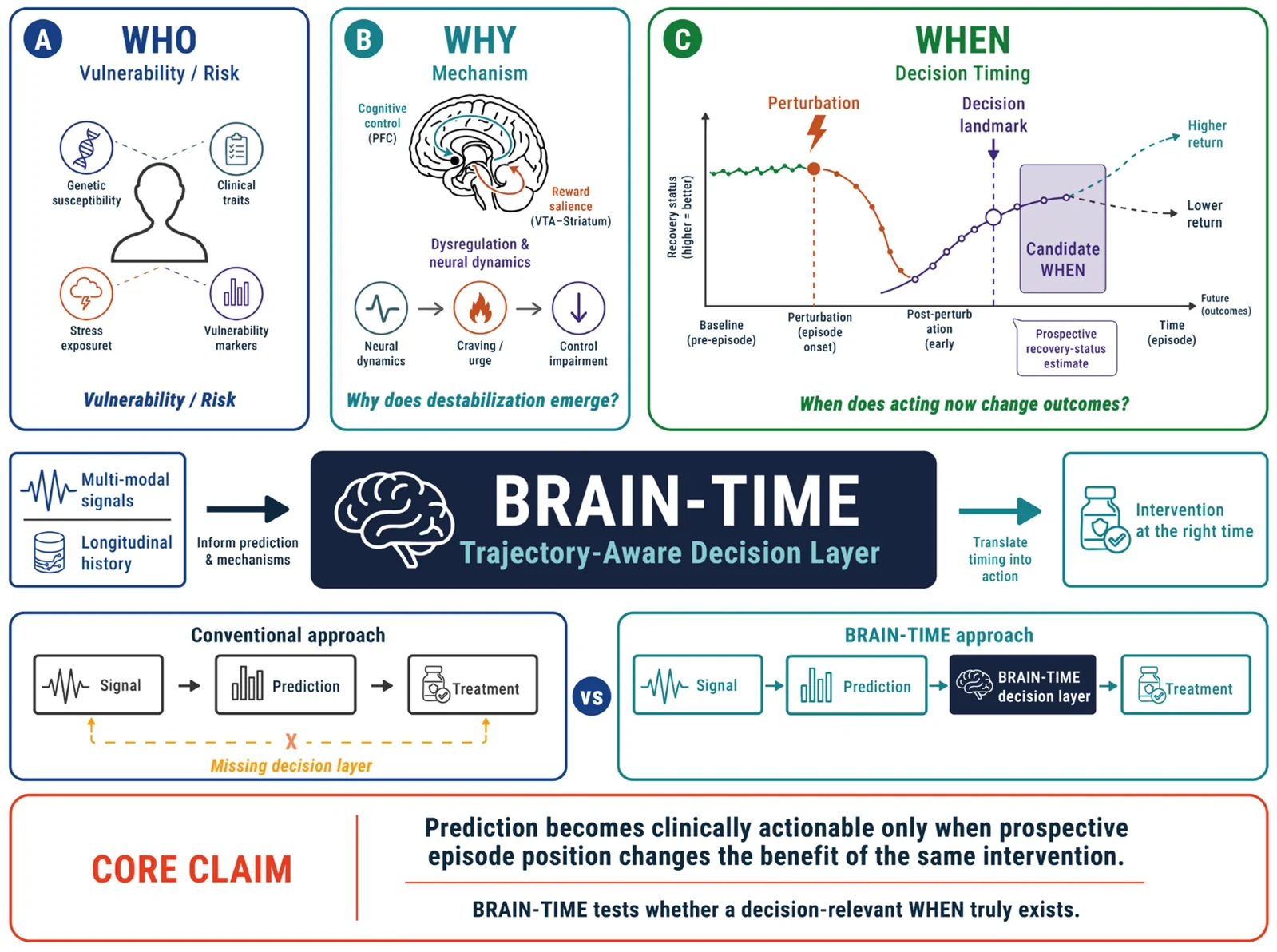
The WHO–WHY–WHEN research programme and the BRAIN-TIME decision layer. Panel (**A**) (WHO) summarizes person-level vulnerability and regulatory-risk stratification addressed by MAC/MAB–RCS [15]. Panel (**B**) (WHY) summarizes the mechanistic level addressed by Reward Instability Theory [16]. Panel (**C**) (WHEN) depicts the BRAIN-TIME episode architecture: a defined perturbation, an evolving post-perturbation trajectory, a locked decision landmark, and a prospectively estimated episode position evaluated as a candidate WHEN. The lower panels contrast the conventional signal–prediction–treatment pathway with the BRAIN-TIME pathway, which inserts an explicit decision layer between prediction and treatment. The central claim is that prediction alone does not establish WHEN; decision relevance requires evidence that prospectively estimated episode position modifies the benefit of the same intervention.

Early-warning systems and JITAIs are not competitors to be dismissed; they represent established adjacent paradigms against which the incremental contribution of BRAIN-TIME must be clearly specified. Neither paradigm, by definition, requires the complete evidentiary sequence proposed here: a defined perturbation, adjudication of sustained return or censoring, leakage-restricted prospective estimation of recovery position, and demonstration that this position modifies the effect of the same intervention. BRAIN-TIME therefore does not replace prediction or adaptive-intervention frameworks; it inserts an additional evidentiary question between prospective information and treatment timing. Section Novelty Relative to Adaptive-Intervention and Causal Machine-Learning Approaches specifies this contribution in terms of target estimands, required designs, and causal assumptions, relative to dynamic treatment regimes, sequential multiple assignment randomized trials, just-in-time adaptive interventions, and causal machine learning. Figure 1 summarizes the distinction among vulnerability, mechanism, and treatment timing and links it to the episode architecture developed in Section 7.

## 4. From Prediction to a Decision-Relevant Transition

### 4.1. Prediction Does Not Determine Action

Physiological and digital monitoring systems can generate informative predictions, but prediction alone does not establish when intervention becomes actionable. A risk estimate may increase while treatment benefit remains unchanged, whereas treatment benefit may vary before a conventional alert threshold is reached.

Apparent divergence of a monitored trajectory from its expected course before overt deterioration may be scientifically informative but is not itself a clinical decision criterion, as it may reflect measurement artefact, model specification, benign adaptation, or temporal structure unrelated to treatment effect [14]. BRAIN-TIME therefore defines a stricter requirement: a prospectively estimated episode position becomes decision-relevant only if the effect of the same intervention differs across positions by a prespecified, practically meaningful amount.

This does not require a discrete biological state or change point. The benefit of intervention may vary smoothly with episode position; a “decision-relevant transition” therefore denotes a region in which the difference in treatment benefit becomes practically important, not an intrinsic discontinuity in the biological process.

### 4.2. Brain-State Dynamics Are Mechanistic Background, Not the Endpoint

Cues, stressors, sleep disruption, and affective challenges can alter reward, salience, cognitive-control, interoceptive, and autonomic processes [18], while neural activity unfolds across heterogeneous timescales and dynamically reconfigures across networks [12,13]. These dynamics make temporal heterogeneity biologically plausible, but no network configuration, dynamical transition, or biomarker is itself a treatment window.

**Figure 2:**
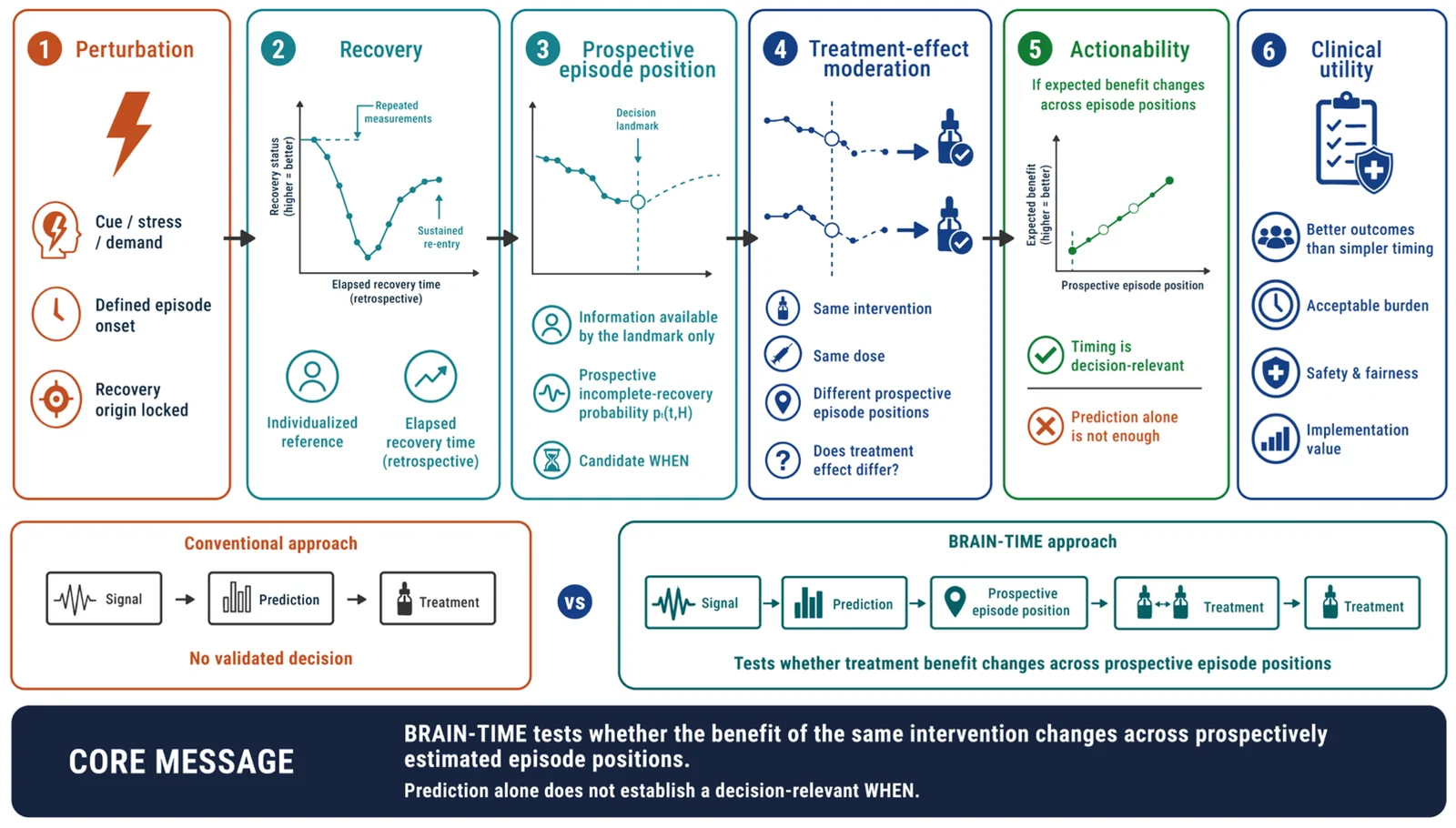
The BRAIN-TIME validation pathway from perturbation to decision utility. Panel (**1**) shows the defined perturbation and locked recovery origin. Panel (**2**) depicts the retrospectively adjudicated recovery trajectory relative to an individualized reference, including sustained re-entry or censoring. Panel (**3**) shows the candidate WHEN as a prospective episode-position estimate based only on information available at the locked decision landmark. Panel (**4**) shows the Stage 1 test, in which the effect of the same intervention and dose is compared across episodes occupying different prospectively estimated positions. Panel (**5**) defines temporal actionability: prospective episode position becomes decision-relevant only if treatment benefit varies across positions by a prespecified, practically meaningful amount. Panel (**6**) represents the subsequent clinical-utility stage, in which a recovery-informed timing strategy must still demonstrate benefit relative to prespecified alternatives. The lower panels contrast the conventional signal–prediction–treatment pathway with the additional decision layer introduced by BRAIN-TIME. The central claim is that prospective recovery information justifies timing-policy testing only if it first modifies the benefit of the same intervention.

BRAIN-TIME therefore uses dynamical neuroscience as mechanistic background rather than as the decision endpoint. Unlike Reward Instability Theory [16], it does not propose another account of reward-system instability; instead, it asks whether prospectively estimated position within a perturbation-anchored trajectory modifies the effect of a fixed intervention. Figure 2 places this question within the cumulative validation pathway from measurement and prospective estimation to temporal actionability and, subsequently, clinical utility.

## 5. Addiction as a Perturbation–Trajectory–Decision System

Addiction is well suited to testing whether temporal information can become decision-relevant because clinically salient perturbations, evolving within-person responses, and experimentally standardized interventions can be studied within the same domain. In BRAIN-TIME, recovery kinetics refers to observable properties of a post-perturbation trajectory—such as rate of return, persistence, rebound, stability, or failure to achieve sustained re-entry—rather than to a latent state or universal recovery programme. The key question is whether such trajectories can support prospective estimation of episode position and, ultimately, identify variation in treatment benefit.

### 5.1. Perturbations and Mechanistic Heterogeneity

Drug-related cues, stress, negative affect, sleep loss, interpersonal conflict, and changes in access to rewarding alternatives can provide identifiable perturbations around which addiction-related trajectories may be organized. Ecological momentary data demonstrate temporally structured relationships among cues, craving, and substance use [18], while early-treatment craving trajectories vary substantially between individuals and may carry long-term prognostic information [19]. Mechanistic studies further support heterogeneous perturbation responses: opioid exposure produces generalized dopamine-neuron cue reactivity [20], conditioned accumbal dopamine transients can forecast individual preference for drug over natural rewards and compulsive behavior [21], and human postmortem multiomic studies reveal cell-type-specific molecular and regulatory heterogeneity in alcohol and cocaine use disorders [22,23]. Together, these findings support the plausibility of individual variation in both perturbation responses and subsequent recovery kinetics.

Clinical heterogeneity may further shape these trajectories without defining the BRAIN-TIME construct itself. Traumatic brain injury provides one example: in an outpatient opioid use disorder sample, nearly half of participants reported a history of TBI, which more often preceded than followed first opioid use [24]. Depression, post-traumatic stress disorder, chronic pain, and other co-occurring conditions may likewise influence perturbation sensitivity, trajectory shape, recovery kinetics, or treatment response. Such factors should therefore be treated as prespecified candidate modifiers or covariates rather than assumed determinants of recovery position.

### 5.2. Craving as a Prospectively Trackable Trajectory, Not Only a Predictor

Craving provides a particularly tractable candidate recovery domain because it can be sampled repeatedly at a temporal resolution unavailable to conventional cross-sectional assessment. Intensive longitudinal research demonstrates time-structured relationships among craving, affective states, substance use, and contextual variables [25], while individual differences in early-treatment craving dynamics have been associated with substance-use status five or more years later [19]. These findings suggest that trajectory shape may contain information beyond a single symptom measurement: the same craving score may occur during rapid resolution, persistent displacement, plateau, rebound, or unstable return.

Such prognostic information should not be equated with the prospective BRAIN-TIME quantity. A trajectory feature may predict a later outcome while depending on retrospective information or carrying no relevance to treatment effect. Recovery position must therefore be estimated using only information available at the locked decision landmark, without future-information leakage. Its decision relevance is then tested separately through treatment-effect modification, as formalized in Section 8 and Section 9.

### 5.3. Why Standardized Interventions Create a Testable WHEN Question

Testing treatment-effect modification by recovery position requires an intervention whose identity and delivery can be sufficiently standardized. Existing addiction studies demonstrate that this is feasible across mechanistically distinct modalities, although they do not imply a common recovery process. Examples include sham-controlled rTMS for stimulant use disorders [26], standardized psilocybin-assisted treatment in alcohol use disorder [27], a prespecified semaglutide regimen in alcohol use disorder [28], a GLP-1 receptor agonist protocol combining structured treatment exposure with repeated craving assessment in opioid use disorder [29], and acute aerobic exercise in methamphetamine use disorder [30]. A longer-duration exercise trial in cannabis use disorder further demonstrates that standardized experimental exposure can yield informative null findings [31]. Collectively, these studies show that controlled intervention exposure, prospective outcome measurement, repeated sampling, and both positive and null results are achievable in addiction research.

For BRAIN-TIME, intervention identity, dose, delivery format, comparator, eligibility criteria, decision landmark, and outcome window must be prespecified. At a common locked landmark, intervention versus comparator is randomized, while prospective recovery position is evaluated as an effect modifier. The resulting treatment-by-position test asks whether the effect of the same intervention varies meaningfully across prospectively estimated recovery positions. This establishes treatment-effect heterogeneity, not yet a timing policy; the latter requires the subsequent policy-level test developed in Section 9.

### 5.4. Independence of the Outward-Function Criterion

The primary BRAIN-TIME trajectory is restricted to one prespecified domain, such as cue-evoked craving, autonomic arousal, attentional capture, or another validated neural or behavioral measure. Collapsing domains with different biological timescales into a single composite could obscure delayed or unstable recovery in one component. Recovery should therefore be adjudicated within the selected domain relative to an individualized reference and sustained-return criterion, while other signals may serve as covariates, controls, or secondary outcomes.

Outward function serves a separate inferential role. Preserved abstinence, task completion, attendance, treatment engagement, or behavioral regulation may coexist with continued displacement in the primary recovery domain. The outward-function criterion should therefore remain conceptually and operationally independent of the recovery trajectory and should not be incorporated into the prospective incomplete-recovery probability. Episodes in which outward function has already deteriorated no longer instantiate the intended preserved-function dissociation and should be excluded or separately flagged according to prespecified rules.

## 6. What Recent Evidence Adds—And What It Does Not

Recent studies provide complementary but heterogeneous evidence that temporally organized neural and physiological information can be measured prospectively and may influence clinically relevant outcomes. This literature includes a transition between distinct dynamical regimes in rat frontal cortex and striatum during perceptual decision formation [32], theta-synchronized closed-loop stimulation that enhanced cortico-hippocampal connectivity in humans relative to phase-blind stimulation [33], and auditory cue exposure delivered during the UP-state of slow-wave sleep that reduced craving and gaming time in internet gaming disorder relative to comparable exposure during wakefulness [34].

Addiction-specific studies further indicate that cue-evoked ventral nucleus accumbens electrophysiology may inform DBS reprogramming for craving suppression in opioid use disorder [35], that Neurobiological Craving Signature expression is associated with craving, disorder severity, and subsequent alcohol use and relapse-related outcomes [36], and that experimentally induced psychosocial stress and cue reactivity are associated with later ambulatory craving and alcohol consumption [37].

Collectively, these findings support the measurability and potential clinical relevance of neural dynamics, temporal alignment, physiological state, and cue- or stress-responsive signals. The sleep-versus-wake study [34] comes closest to the causal logic motivating BRAIN-TIME because intervention effects differed by physiological state, whereas theta-locked stimulation [33] demonstrates that temporal alignment can causally alter human network physiology. However, the cited studies address distinct quantities—neural regime transition [32], oscillatory phase [33], sleep–wake state [34], cue-reactive electrophysiology [35], neuroimaging signature expression [36], and stress response [37]—and none defines an individualized post-perturbation recovery trajectory, retrospectively adjudicates sustained re-entry relative to an individualized reference, or prospectively estimates recovery position at a locked decision landmark.

BRAIN-TIME therefore treats this literature as evidence of mechanistic plausibility and empirical feasibility rather than direct validation. The unresolved question is whether a prospectively estimated position within an individualized recovery process modifies the benefit of the same intervention. Establishing that link requires interpretable recovery kinetics, leakage-restricted prospective estimation at a locked decision landmark, and randomized treatment-effect moderation. Until those conditions are met, the available evidence supports the plausibility of the WHEN question but does not answer it.

## 7. The Minimal BRAIN-TIME Episode Architecture

Section 1, Section 2, Section 3, Section 4, Section 5 and Section 6 establish the conceptual boundary of the WHEN question: it is distinct from vulnerability and mechanism, cannot be inferred from prediction alone, is experimentally tractable in addiction, and is not yet answered by the closest state-dependent, closed-loop, or trajectory-based evidence. The next step is therefore not to introduce another descriptive construct, but to reduce the framework to a specification precise enough to be falsified. The purpose of this formalization is translational rather than methodological in isolation: it specifies the evidentiary conditions under which an addiction-related mechanistic trajectory could legitimately inform a treatment decision. BRAIN-TIME is deliberately restricted to four formal quantities: the observed post-perturbation trajectory, retrospectively adjudicated elapsed recovery time, prospective probability of incomplete recovery, and recovery-position-dependent treatment effect. Recovery kinetics—including rate of return, persistence, rebound, residual displacement, and stability—describes properties of the observed trajectory but is not introduced as a fifth latent construct. This restriction prevents BRAIN-TIME from becoming an all-purpose state model and preserves a strict inferential sequence from trajectory measurement to recovery adjudication, prospective estimation, treatment-effect moderation, and only subsequently policy-level timing evaluation. Table 3 defines these quantities and, critically, specifies what each can and cannot establish independently. The term recovery position is a decision-oriented shorthand for the prospectively estimated probability that recovery will remain incomplete beyond a prespecified horizon under the specified background policy; it is not assumed to represent an ordinal or geometrically unique coordinate on the underlying biological trajectory.

These quantities form an inferential sequence rather than alternative labels for the same biological state. Narrative expressions used elsewhere in the article—recovery position, episode position, candidate WHEN, and decision-relevant transition—therefore denote different evidentiary interpretations of the prospective incomplete-recovery quantity rather than additional formal constructs. A candidate WHEN is a value or region of prospective episode position selected for evaluation. It becomes decision-relevant only if the prespecified treatment-by-recovery-position interaction defined in Section 9 and operationalized in Table 4 exceeds a prespecified minimum practically important magnitude. The corresponding Stage 1 prespecification framework is operationalized in Supplementary Table S1. Accordingly, trajectory structure and recovery kinetics motivate the temporal hypothesis, prospective estimation locates the candidate decision position, and randomized treatment-effect moderation determines whether that position is decision-relevant. None of the preceding quantities is intrinsically actionable.

### 7.1. Qualifying Perturbation and Recovery Origin

A qualifying perturbation must be temporally identifiable and characterized with sufficient precision to permit reproducible episode definition. Its intensity, duration, repetition, context, prior exposure, sleep state, medication status, and relevant co-exposures should be prespecified where applicable. Controlled, low-risk perturbations are particularly appropriate for initial construct testing because both episode origin and exposure characteristics can be experimentally constrained; naturalistic perturbations are better reserved for subsequent transportability testing. The recovery origin is ordinarily defined as the end of the perturbation unless an alternative biologically justified offset is specified independently of the subsequent trajectory. Critically, the recovery origin must be locked without reference to how rapidly, slowly, or completely the episode subsequently resolves.

All initiated qualifying episodes must enter participant flow. Restricting analysis to episodes displaying large craving responses, prolonged displacement, or visually apparent recovery patterns would condition inclusion on the phenomenon the framework is intended to detect and could manufacture apparent recovery structure. Episodes should instead be classified according to prespecified rules as non-displacing, evaluable, interval-censored, right-censored, affected by a prespecified intercurrent or competing event, or uninterpretable. A non-displacing episode is itself informative: it indicates that the qualifying perturbation did not produce sufficient displacement in the prespecified domain to support estimation of recovery kinetics for that episode and should not be retrospectively relabelled to preserve an anticipated effect.

### 7.2. Sustained Re-Entry

For participant *i*, recovery domain *d*, and episode *e*, elapsed recovery time is defined as the earliest post-perturbation time at which the observed trajectory achieves sustained re-entry into the prespecified individualized restoration set:

$$T_{i,d,e}^{R}=\inf\left\{\tau \geq 0:X_{i,d,e}\left(t_{0,i,d,e}+\tau +s\right)\in R_{i,d}\left(t_{0,i,d,e}+\tau +s\right)\;\forall s\in \left[0,\delta \right]\right\}$$

Here, $T_{i,d,e}^{R}$ denotes elapsed recovery time; *t*_0,*i*,*d*,*e*_ is the locked recovery origin; *X_i_*_,*d*,*e*_(t) is the observed post-perturbation trajectory in the prespecified primary recovery domain; ℛ*_i_*_,*d*_(*t*) is the prespecified phase- and context-aware individualized restoration set; *τ* denotes elapsed time from the recovery origin; and *δ* is the prespecified stability interval required to establish sustained rather than transient re-entry. Recovery is therefore not defined by the first threshold crossing: transient normalization followed by renewed displacement, rebound, or unstable return remains part of the observed recovery kinetics until the sustained-re-entry criterion is satisfied. The restoration set must be specified independently of the realized episode outcome and remain conceptually distinct from uncertainty in the prospective model; greater uncertainty may widen uncertainty intervals, reduce interpretability, or trigger a prespecified no-interpretation output, but it must not make recovery easier to declare by retrospectively relaxing, shifting, or reconstructing the restoration criterion. If sustained re-entry is never achieved, the defining set is empty and the underlying recovery time is conceptually unbounded; under finite follow-up, however, the observed endpoint is right-censored rather than assigned an arbitrary terminal recovery time. This distinction preserves the explicitly retrospective role of $T_{i,d,e}^{R}$: it adjudicates what ultimately occurred during the episode but cannot represent what was knowable at an earlier decision landmark. The recovery endpoint defined here therefore provides the outcome against which prospective estimation can subsequently be developed and evaluated, while remaining formally distinct from the decision-time recovery-position quantity introduced in Section 8.

### 7.3. Censoring, Competing Events, and Intercurrent Events

Under discrete observation, the exact onset of sustained re-entry is generally localized to an interval rather than observed directly. The recovery endpoint is therefore interval-censored, and if sustained re-entry is not demonstrated before the prespecified observation horizon, the observed recovery time is right-censored rather than assigned an arbitrary terminal value. Joint longitudinal–time-to-event modelling provides one principled analytical family for future implementation, although the framework does not depend on any single model class [38].

Censoring must not, however, become a catch-all category: a competing event precludes the event of interest, whereas censoring denotes incomplete observation of an endpoint that could otherwise still occur [39], and intercurrent events in the sense of ICH E9(R1) [40] affect the interpretation or the existence of measurements relevant to the clinical question. Clinically distinct post-perturbation events should therefore be mapped prospectively to estimand-specific roles rather than pooled by analytical convenience (Supplementary Materials, Section S2).

## 8. The Prospective WHEN Quantity

Completed recovery is unavailable at the moment at which a treatment decision must be made. The decision-time quantity must therefore be formally distinct from retrospectively adjudicated recovery time and computable exclusively from information available by a prespecified, locked decision landmark. For participant *i*, recovery domain *d*, episode *e*, landmark *t*, and prespecified future horizon *H*, the prospective incomplete-recovery probability is defined conceptually as

$$p_{i,d,e}\left(t,H\right)=P\left[T_{i,d,e}^{R}\left(\pi_{0}\right)>\left(t-t_{0,i,d,e}\right)+H|I_{i,d,e}\left(t\right)\right]$$

Here, *p_i_*_,*d*,*e*_(*t*,*H*) denotes the probability, evaluated at the decision landmark, that recovery will remain incomplete beyond the next *H* units of time; *t*_0,*i*,*d*,*e*_ is the locked recovery origin; *I_i_*_,*d*,*e*_(*t*) is the information set containing only data legitimately available by landmark *t*; and $T_{i,d,e}^{R}\left(\pi_{0}\right)$ denotes the potential elapsed recovery time under the prespecified background treatment or support policy *π*_0_. The background policy excludes the additional randomized Stage 1 intervention evaluated in Section 9. For readability, the domain and episode indices are subsequently suppressed, yielding

$$p_{i}\left(t,H\right)=P\left[T_{i}^{R}\left(\pi_{0}\right)>\left(t-t_{0,i}\right)+H|I_{i}\left(t\right)\right]$$

This formulation deliberately separates the target quantity from the model used to estimate it. This separation follows the estimand logic formalized in ICH E9(R1) [40]. Because *p_i_*(*t*,*H*) concerns a future recovery outcome under a specified background policy rather than merely the factual outcome observed under whatever treatment subsequently occurs, its estimation constitutes a counterfactual-prediction problem. Counterfactual prediction requires explicit assumptions connecting observed data to outcomes under the target policy and requires performance to be evaluated for the policy-specific quantity actually being predicted rather than inferred from ordinary factual prediction alone [41].

This distinction is central to BRAIN-TIME because an intervention administered after the landmark may itself alter subsequent recovery. Post-randomization observations must therefore not be allowed to redefine the prospective position used to evaluate treatment-effect heterogeneity. Separately, estimation depends on a development population compatible with the target background policy, adequate support across the recovery-position distribution, and explicit transport assumptions. Failure of these conditions represents a limitation of identification, estimation, or transportability; it is not evidence for or against the biological existence of recovery dynamics.

The prospective recovery-position model should be developed and calibrated in a designated development set, or in otherwise appropriately held-out episodes, and then locked before confirmatory treatment-effect testing. This separation is operationalized in Supplementary Table S1.

Counterfactual prediction defines which future quantity is being estimated, whereas dynamic prediction addresses how that estimate is updated as longitudinal information accumulates before the decision landmark. Methods for dynamic event prediction using longitudinal markers therefore provide a complementary methodological basis for repeatedly estimating *p_i_*(*t*,*H*) as new observations become available [42]. Landmark-based dynamic survival analysis provides one explicit framework for enforcing the restriction of each estimate to the information set available at its own landmark, and for comparing more complex trajectory models with simpler alternatives [43]. Model development based on complete longitudinal histories can inadvertently expose an algorithm to observations that would not have been available prospectively. In BRAIN-TIME, such leakage would not merely inflate conventional predictive performance; it would compromise the definition of the candidate WHEN itself by transforming a retrospectively recognizable trajectory into an apparently prospective decision variable.

Prediction-model development, evaluation, appraisal, and reporting constitute distinct inferential tasks. The Transparent Reporting of a multivariable prediction model for Individual Prognosis Or Diagnosis + Artificial Intelligence (TRIPOD+AI) statement provides guidance for transparent reporting of prediction-model studies using regression or machine-learning methods [44], whereas the Prediction model Risk Of Bias ASsessment Tool + Artificial Intelligence (PROBAST+AI) addresses methodological quality, risk of bias, and applicability [45]. Predictive performance should be reported across calibration, discrimination, and decision-analytic dimensions rather than collapsed into a single measure of accuracy [46]; the full set of required dimensions is specified in Supplementary Section S3. Relative to prespecified simpler benchmarks, if elapsed time or current state provides equivalent prospective information and equivalent downstream treatment-effect-modification information, the rationale for introducing a more complex recovery-position construct is correspondingly weakened.

Uncertainty requires an additional safeguard, because *p_i_*(*t*,*H*) is an estimated quantity rather than an observed biological state. That uncertainty, whose sources are enumerated in Supplementary Section S3, should therefore be propagated into, or otherwise explicitly accounted for in, the subsequent Stage 1 treatment-effect-modification analysis. The uncertainty-handling procedure should consequently be prespecified rather than selected after the treatment-effect result is known. Coverage, abstention, and no-interpretation reporting requirements are likewise specified in Supplementary Section S3.

Early-warning research illustrates that a signal may precede deterioration without establishing that intervention on the signal changes outcome, while intervention triggered by a warning may itself alter the outcome against which predictive performance is subsequently evaluated [47]. An AI-enabled deterioration intervention embedded within a defined threshold-triggered response pathway has been associated with improved downstream clinical outcomes [48]. Such evidence supports the specific model–threshold–workflow–response combination that was evaluated; it does not establish that another predictor with comparable statistical performance would produce equivalent benefit when repurposed to determine treatment timing. BRAIN-TIME consequently treats prediction and actionability as sequential evidentiary achievements: predictive validity is required for a defensible prospective recovery-position quantity, but predictive validity does not establish a WHEN.

The prospective incomplete-recovery probability consequently remains a decision-time information quantity, not a treatment indication, biological state label, or timing rule. Section 9 provides the decisive Stage 1 causal test.

## 9. The Decisive Test: Does the Same Intervention Work Differently Now?

The decisive translational claim of BRAIN-TIME is not that an addiction-related recovery trajectory can be described or predicted, but that prospectively available recovery information may identify clinically meaningful variation in treatment benefit that can be tested causally. Stage 1 must therefore separate recovery position from treatment selection. BRAIN-TIME asks whether prospective recovery position modifies the effect of a fixed intervention before it asks whether different interventions should be selected at different positions.

At the common locked decision landmark, the active intervention and comparator are randomized independently of estimated recovery position, while prospective recovery position enters the confirmatory analysis as a prespecified effect modifier. Let Y_i_(1) denote the potential primary outcome for participant or episode *i* under the active intervention and Y_i_(0) the corresponding potential outcome under the comparator. Conceptually, Stage 1 evaluates the conditional treatment-benefit function

Δ(p) = E{Y_i_(1) − Y_i_ (0)∣p_i_ (t,H) = p}

Here, Δ(p) denotes the expected benefit of the active intervention relative to the comparator at recovery position p = p_i_(t,*H*), as defined in Section 8. The outcome scale, coding, and treatment-effect sign convention should be specified prospectively so that the direction of benefit is unambiguous. The purpose of this function is not to identify an intrinsic threshold or preferred treatment time, but to represent whether the expected benefit of the same randomized intervention changes across prospectively estimable recovery positions.

The conditional treatment-benefit function should not itself be conflated with the confirmatory Stage 1 estimand. Rather, the confirmatory estimand should be a prospectively defined functional of Δ(p) that quantifies clinically meaningful variation in treatment benefit across the recovery-position distribution. The corresponding operational prespecification requirements are detailed in Supplementary Table S1.

Prospective recovery position should remain continuous in the primary analysis whenever feasible; the admissible uses of prespecified contrasts and operational strata are specified in Supplementary Section S4. Such categories should not replace the continuous primary quantity or be interpreted as natural recovery states, latent phenotypes, biological phases, or treatment indications. Data-driven thresholds, post hoc subgroup searches, and retrospective selection of apparently responsive regions cannot establish temporal actionability.

Because Stage 1 targets treatment-effect heterogeneity rather than the marginal treatment effect, design and information planning must target that heterogeneity directly. A study adequately powered for an average intervention effect may remain insufficiently informative for the central BRAIN-TIME hypothesis. Uncertainty in recovery position must likewise remain visible, because the moderator is estimated rather than directly observed; the requirements for support across the recovery-position distribution and for handling moderator uncertainty are specified in Supplementary Section S4.

A further safeguard is required when the same clinical domain contributes both to recovery-position estimation and to the post-intervention outcome. If craving trajectories, for example, inform prospective recovery position and craving also contributes to the primary outcome, an apparent treatment-by-position effect may partly reflect current symptom severity, regression to the mean, shared measurement structure, or mathematical coupling rather than incremental temporal information carried by recovery dynamics. The analysis should therefore prespecify comparison with the current landmark value of the same domain and evaluate whether treatment-effect modification by recovery position persists beyond treatment-effect modification by current state alone. If the apparent recovery-position effect is fully reproduced by current landmark severity, the claim that trajectory-derived recovery information contributes additional temporal decision value is substantially weakened or removed.

The interpretation of a non-positive Stage 1 result must depend on whether the target claim was evaluated with adequate information. An adequately informative and sufficiently precise null or practically negligible treatment-by-recovery-position effect constitutes a meaningful falsification of temporal actionability for the tested intervention and context. Such a result constitutes a STOP for that intervention-specific timing hypothesis, whereas a target claim that could not be adequately evaluated yields an INCONCLUSIVE classification rather than falsification. Conversely, prospective recovery position need not be an exceptionally strong general prognostic marker to become decision-relevant if it reproducibly identifies practically important variation in the effect of a specified intervention.

A subsequent Stage 2 experiment must test the timing-policy question directly. One approach is to randomize participants, or eligible episodes when intervention effects are sufficiently proximal and carryover can be adequately controlled, between a fixed-schedule policy and a prospectively defined recovery-informed delivery policy. In settings with repeated decision opportunities, intervention availability may instead be randomized across multiple prespecified landmarks using a micro-randomized design, allowing time-varying treatment effects and their moderation by decision-time context to be evaluated through causal excursion effects [17,49]. Such designs shift the inferential target from whether recovery position contains treatment-effect information to whether acting on that information improves patient-important outcomes. Any recovery-informed action rule derived from Stage 1 evidence should therefore be specified prospectively and locked before Stage 2 outcomes are observed rather than reconstructed retrospectively from the policy trial itself. The Stage 2 estimand is consequently a policy-level outcome contrast, not the Stage 1 conditional treatment-benefit relationship.

The evidentiary sequence is therefore deliberately asymmetric: prediction may justify testing treatment-effect modification, and treatment-effect modification may justify testing a timing policy, but neither mechanistic plausibility nor predictive performance can substitute for the causal test required at the next stage. In BRAIN-TIME, retrospective recovery establishes what occurred; prospective estimation determines what can legitimately be known at the decision point; Stage 1 determines whether that information modifies expected treatment benefit; and Stage 2 determines whether acting on it improves patient-important outcomes.

## 10. Minimal Proof of Concept in Addiction

The initial BRAIN-TIME study should be a mechanism-to-treatment proof of concept rather than a trial of clinical utility or a recovery-informed timing policy. Its purpose is to test the complete Stage 1 chain under prospectively controlled conditions while minimizing degrees of freedom by prespecifying one target substance use disorder and treatment context, one perturbation class, one primary recovery domain, one decision landmark, one intervention, one comparator, and one primary outcome (Table 4). This restriction limits construct proliferation, treatment heterogeneity, temporal leakage, multiplicity, and post-outcome model adaptation.

### 10.1. Design Requirements and Interpretation of the First Proof of Concept

The first proof of concept should not use relapse as its sole primary endpoint. Although clinically important, relapse is relatively infrequent over short experimental windows and is strongly influenced by background treatment, social context, substance availability, and environmental exposure. A more appropriate initial endpoint is therefore a prespecified proximal, clinically interpretable consequence of intervention, such as craving-related interference, distress burden, coping success, functional engagement, or another recovery-relevant outcome measured within a defined window.

Participant safety is part of the scientific validity of the first study. Challenge intensity and duration, participant- and investigator-initiated stopping rules, post-session observation, escalation procedures, and exclusion criteria for participants at elevated risk should be prespecified and reviewed by the responsible ethics committee or institutional review board before enrollment. Poorly standardized provocation would also compromise inference by increasing rescue interventions and other intercurrent events and by selectively removing the most reactive episodes from observation.

The first proof of concept should ultimately answer one narrow question: does prospectively estimated recovery position modify the benefit of the same randomized intervention, delivered at the same locked decision landmark, by a prespecified practically meaningful amount?

### 10.2. A Worked Example of the Minimal Stage 1 Specification

The following hypothetical study shows how the elements of Table 4 and the requirements set out above combine in a single concrete design. It is not a recommendation for a particular intervention or measure. Ninety clinically stable outpatients with alcohol use disorder, receiving a defined and stable background support policy, each contribute two episodes separated by at least seven days, yielding 180 episodes. Participants with acute intoxication, clinically significant withdrawal, or any prespecified safety exclusion are not enrolled.

The perturbation is a single standardized low-intensity alcohol-cue challenge of four minutes, delivered at a fixed time of day, with a defined intensity range, a recovery origin set at cue offset, prespecified termination criteria, and an unconditional participant- or investigator-initiated stopping rule. The primary recovery domain is self-reported urge to drink, sampled every five minutes. The individualized reference is each participant’s mean urge across a dense pre-perturbation baseline day, estimated independently of the confirmatory episode and locked before recovery adjudication. Recovery is adjudicated as sustained re-entry into the individualized reference range maintained for twenty continuous minutes, with an explicit recurrence rule and prespecified handling of interval censoring.

The independent outward-function criterion is verified task engagement on a brief concurrent attention task, operationally non-overlapping with the urge trajectory used to estimate recovery position. The decision landmark is fixed at forty-five minutes after cue offset, defined independently of the subsequent trajectory.

The prospective quantity is the locked continuous probability that sustained re-entry will not occur within the following sixty minutes, generated from the urge observations available by minute forty-five together with baseline features, under the background policy. A no-interpretation output is returned when fewer than seven of the nine scheduled observations are usable, when the episode falls outside the supported development region, or when the individual prediction interval exceeds a prespecified width. The target coverage is 85 percent of eligible episodes.

At the landmark, episodes are randomized one-to-one between a brief twenty-minute guided regulation module and an attention-, time-, and staff-contact-matched comparator, with fixed content and monitored fidelity held constant across recovery positions. Randomization is stratified by three operational recovery-position classes while the continuous quantity remains the primary effect modifier.

The primary outcome is craving-related interference with the concurrent task, assessed in a locked window from sixty to ninety minutes after randomization, with the direction of benefit and sign convention fixed before analysis. Because both the recovery domain and the primary outcome draw on craving, the current-state safeguard applies: the confirmatory analysis prespecifies comparison with the urge value observed at minute forty-five and evaluates whether effect modification by recovery position persists beyond effect modification by current urge alone.

The primary estimand is the difference in expected treatment benefit between recovery positions of 0.25 and 0.75, with a minimum practically important criterion of 0.30 standard deviations on the outcome scale. A GO classification requires that gates 1–4 in Table 5 have been satisfied, that the estimated difference exceeds 0.30 standard deviations with adequate precision, and that realized coverage and treatment-arm overlap meet their prespecified minima.

A precise estimate of 0.05 standard deviations with an interval excluding 0.30 constitutes a STOP for this intervention and context. A point estimate of 0.35 standard deviations with an interval spanning zero, or realized coverage of 62 percent, yields an INCONCLUSIVE classification. The former calls for additional information, the latter for refinement of the prospective estimation procedure, and neither warrants progression to a timing-policy trial.

## 11. Falsification, Uncertainty, and Boundary Conditions

BRAIN-TIME is designed to be falsifiable at explicitly separated inferential levels. Table 5 organizes the framework into six cumulative, non-compensatory evidence gates: success at a later stage cannot validate failure or unresolved evidence at an earlier one. Failure should be interpreted at the level of the claim tested; failure of a particular sensor, recovery domain, perturbation paradigm, prediction model, or intervention constrains that implementation rather than automatically invalidating the framework as a whole.

Gate failure should be concluded only when the relevant claim has been evaluated with sufficient information; limitations that prevent a defensible test yield an INCONCLUSIVE rather than a STOP classification. Supplementary Table S2 operationalizes GO, STOP, and INCONCLUSIVE classifications without adding further evidence gates.

Uncertainty must remain explicit throughout the evidentiary sequence. Restricting analysis to high-confidence episodes is not a neutral solution because it changes the target population and may introduce selection. Uncertainty should consequently contribute directly to GO, STOP, or INCONCLUSIVE classification.

The preserved-function condition requires particular caution. Conditioning confirmatory analyses on preserved outward function may introduce selection or collider-related bias if preservation is jointly influenced by recovery-related processes and other determinants of treatment response or outcome [50]. Its role should therefore be defined prospectively and represented explicitly in the causal structure, with the required sensitivity analyses specified in Supplementary Section S5.

Signal classes should also remain construct-specific. Neural, autonomic, behavioral, and subjective trajectories may evolve on different timescales and should not be assumed to corroborate one another simply because they are recorded during the same episode [12,13]. This is especially important for wearable autonomic measures: PPG-derived measurements may differ materially from ECG references [51], and HRV interpretation is strongly context-dependent [52]; the specific dependencies and the resulting validation requirements are detailed in Supplementary Section S5. No wearable-derived feature should therefore be treated as a generic surrogate for autonomic recovery, nor should any neural or physiological signal be designated a brain-recovery biomarker without construct-specific validation.

Observed return trajectories may also reflect processes other than restoration, such as habituation, learning, medication effects, or drift in the measurement itself (Supplementary Section S5). If restoration cannot be distinguished from adaptation to the experimental procedure itself, a defensible recovery construct has not been established.

The principal boundary condition of BRAIN-TIME is therefore its sequential scope: defined perturbation → trajectory and recovery kinetics → sustained re-entry or censoring → prospective recovery position → Stage 1 treatment-effect modification → Stage 2 timing-policy utility.

Each transition requires its own empirical support.

## 12. Limitations

BRAIN-TIME is a conceptual and unvalidated architecture rather than an empirical finding, and its claims must be interpreted accordingly. No original data are reported, and every quantity specified in Section 7, Section 8, Section 9 and Section 10 represents a proposed construct, estimand, design requirement, or falsification criterion rather than an observed result. The feasibility of reconstructing sufficiently reliable perturbation-anchored trajectories, the calibration and transportability of prospective recovery-position estimates, the degree of support across the relevant recovery range, and the magnitude of any treatment-by-recovery-position interaction all remain unknown. The framework specifies the evidentiary conditions under which a WHEN claim could become credible; it does not establish that a decision-relevant recovery position exists.

The decisive Stage 1 test presents a substantial information and power challenge, because the target quantity is effect modification across an estimated, continuous moderator rather than a marginal treatment effect. Rigorous evaluation may require repeated-episode sampling, multisite recruitment, or prospectively justified sequential designs rather than reliance on a single conventional fixed-sample experiment.

Ethical constraints impose a second boundary on construct development. The most controlled initial studies are likely to require low-intensity cue or stress challenges in clinically stable participants, with strict stopping criteria, post-challenge monitoring, and rescue procedures. Such designs improve perturbation definition and participant safety but may underrepresent the severity, volatility, and contextual complexity of naturally occurring episodes in which recovery-informed treatment timing might ultimately be most consequential. Conversely, more intense or naturalistic perturbations would increase ecological relevance while weakening experimental control and potentially increasing intercurrent events, rescue interventions, missingness, and selection. The initial proof of concept should therefore be interpreted as construct development rather than evidence of transportability to high-risk clinical episodes.

The intervention examples reviewed in Section 5.3 introduce a related limitation. Neuromodulation, psychedelic-assisted treatment, GLP-1 receptor agonism, and exercise differ in mechanism, biological timescale, dosing structure, and therapeutic context. They are included to demonstrate that standardized intervention exposure and prospective outcome measurement are feasible in addiction research, not to imply a common recovery mechanism or universal treatment window. None of the cited studies directly evaluates the BRAIN-TIME treatment-by-recovery-position interaction, and evidence obtained for one intervention cannot be generalized to another without repeating the relevant perturbation, trajectory, prospective-estimation, and causal effect-modification sequence.

The motivating empirical literature also remains heterogeneous and comparatively immature. Section 6 draws deliberately on recent studies spanning neural regimes, oscillatory timing, physiological state, cue-reactive electrophysiology, craving signatures, and stress reactivity. These studies establish that temporally organized neural and behavioral information can be measured and, in some settings, can relate to intervention response, but they do not constitute convergent validation of a common post-perturbation recovery construct. Failure to replicate individual findings would weaken particular mechanistic motivations for prioritizing the programme, although it would not by itself falsify the formal BRAIN-TIME architecture. The framework must stand or fall on its own prespecified evidence gates rather than on the continued plausibility of conceptually adjacent findings.

## 13. Discussion

The principal contribution of BRAIN-TIME is not a new biomarker, predictive algorithm, latent brain state, or general theory of addiction, but an explicit evidentiary layer between prospectively available information and the intervention that information might justify. Its central question is whether the expected benefit of the same intervention varies across prospectively estimated positions within an evolving post-perturbation recovery process.

Recent addiction-specific studies illustrate why this question is not answered by mechanistic or prognostic evidence alone. In a preregistered, randomized, triple-blind, sham-controlled trial in opioid use disorder, theta-band frontoparietal tACS altered cue-related neural activity and functional coupling without significantly changing the prespecified subjective craving outcome [53]. This dissociation shows that mechanistic target engagement does not necessarily translate into clinical benefit. Conversely, task-based EEG connectivity in heroin use disorder supported individualized prediction of cue-induced craving and subsequent improvement after intermittent theta-burst stimulation [54], demonstrating strong prognostic potential without establishing treatment-effect modification by recovery position. Computational work in alcohol drinkers and cannabis users further showed bidirectional interactions between momentary craving and substance-related learning and decision processes [55], supporting a dynamic view of addiction-related states without defining a recovery construct or treatment window. BRAIN-TIME addresses the remaining causal question: whether prospectively estimated position within a defined recovery trajectory modifies the benefit of the same intervention at a common decision landmark.

Decision relevance is therefore relational rather than an intrinsic property of a biomarker or brain state. It depends jointly on the perturbation, trajectory, decision landmark, prospective recovery estimate, intervention, comparator, and outcome. A highly predictive state may be irrelevant to treatment timing if intervention benefit remains constant across it, whereas the same prospective position may be actionable for one intervention but not another. This is why the heterogeneous evidence reviewed in Section 5 and Section 6 can motivate BRAIN-TIME without validating it.

Within the broader research programme, MAC/MAB–RCS addresses WHO through person-level regulatory stratification [15], Reward Instability Theory addresses WHY through the dynamic organization of reward-system dysfunction [16], and BRAIN-TIME addresses WHEN by testing whether prospectively available episode-level information modifies treatment benefit. These frameworks therefore operate at distinct units of analysis and answer distinct inferential questions: susceptibility, mechanism, and temporal decision relevance.

The restrictive architecture of BRAIN-TIME is central to its falsifiability. Elapsed time or current symptom severity may prove sufficient, the recovery-position model may add no incremental information, or an effective intervention may show no meaningful treatment-by-position interaction. Any of these outcomes would constrain or reject the corresponding WHEN claim.

The two-stage architecture—effect-modification testing followed by timing-policy evaluation—is compatible with contemporary Good Clinical Practice. ICH E6(R3) emphasizes prospectively identifying factors critical to trial quality, incorporating quality into study design, and using proportionate risk-based procedures [56]. For BRAIN-TIME, these principles support prespecification of the perturbation, decision landmark, intervention, safety procedures, data-quality safeguards, and progression criteria, but they provide no evidence that a decision-relevant recovery position exists.

The broader translational implication is therefore not to search indiscriminately for hidden treatment windows, but to distinguish mechanism, prediction, and actionability as separate evidentiary achievements. Contemporary addiction neuroscience increasingly provides sophisticated mechanistic and predictive information [53,54,55]. BRAIN-TIME asks whether that information can identify a prospectively knowable episode position at which treatment benefit changes—and makes that claim directly testable rather than assumed.

### Novelty Relative to Adaptive-Intervention and Causal Machine-Learning Approaches

Dynamic treatment regimes, sequential multiple assignment randomized trials, just-in-time adaptive interventions, and causal machine learning all address treatment individualization, and BRAIN-TIME overlaps with each of them in motivation. The distinctions lie in the target estimand, the required design, and the causal assumptions under which the target quantity is identified.

Dynamic treatment regimes target the value of a decision rule: the expected outcome that would be obtained if a specified sequence of treatment decisions were applied to a population, with sequential multiple assignment randomized trials providing the design that permits such rules to be constructed and compared. The estimand is a policy-level contrast, and identification relies on sequential randomization or sequential ignorability together with positivity at each decision point.

BRAIN-TIME Stage 1 does not target a policy value. Its estimand is a functional of the conditional treatment-benefit function at a single locked landmark, and its design randomizes one intervention rather than a sequence. The corresponding BRAIN-TIME policy question is deferred to Stage 2, where a dynamic-regime formulation becomes appropriate; Stage 1 exists precisely to determine whether such a formulation is warranted at all [57].

Just-in-time adaptive interventions and micro-randomized trials target time-varying causal excursion effects and their moderation by decision-time context. The overlap with BRAIN-TIME is genuine, and the difference is in what qualifies as tailoring information. Micro-randomized trials randomize intervention across many decision points and permit decision-time tailoring variables, but do not by themselves establish that a perturbation-anchored, model-derived recovery quantity has been validly constructed and prospectively qualified for that role. BRAIN-TIME requires the moderator to be a prospectively estimated position within a trajectory anchored to a defined perturbation, with a locked recovery origin, an adjudicated sustained-return endpoint or explicit censoring, and a leakage restriction on the information set.

That construction imposes assumptions not required by the micro-randomized framework: that the perturbation is specified and its response separable from background temporal structure, that recovery is adjudicated reproducibly rather than by transient threshold crossing, and that the moderator is computable from information available at its own landmark. These assumptions are what make the resulting quantity a candidate WHEN rather than a context feature, and they are also what render the framework falsifiable at the gates specified in Section 11.

Within the broader precision-medicine programme of deriving individualized treatment rules [58], causal machine learning targets conditional average treatment effects and provides estimation and inference for treatment-effect heterogeneity across high-dimensional covariates [59]. BRAIN-TIME is not an alternative estimator, and the methods of this literature are directly applicable to estimating the conditional treatment-benefit function.

These methods can estimate such heterogeneity conditional on specified covariates or moderators, but do not by themselves validate the temporal construction of a candidate recovery moderator. BRAIN-TIME concerns the prior question of what the moderator may legitimately be: a quantity that is temporally defined, prospectively computable, and estimated with propagated uncertainty, rather than any baseline covariate available in the dataset. Applying a conditional-average-treatment-effect estimator to a retrospectively constructed trajectory feature would reproduce the leakage problem described in Section 8 while retaining the appearance of causal rigor.

The novelty of BRAIN-TIME therefore does not lie in individualizing treatment, in estimating heterogeneous effects, or in adapting intervention timing to context. It lies in specifying the evidentiary requirements a temporal quantity must satisfy before it may be used as the moderator or tailoring variable in any of these frameworks, and in making failure to satisfy them detectable rather than absorbed into model fit.

## 14. Conclusions

Recent addiction neuroscience shows that dynamic craving processes, individualized neural features, and targeted neuromodulation can provide mechanistically and prognostically relevant information [53,54,55]. These advances, however, do not establish whether treatment benefit changes as an episode evolves. BRAIN-TIME addresses this unresolved step by separating trajectory measurement, retrospective recovery adjudication, leakage-restricted prospective estimation, and randomized treatment-effect modification.

The framework asks a narrow, falsifiable question: whether prospectively estimated position within a defined post-perturbation recovery process modifies the benefit of the same intervention delivered at a common locked decision landmark by a prespecified, practically meaningful amount. A GO Stage 1 result would establish treatment-effect heterogeneity and justify testing a recovery-informed timing policy in Stage 2; an adequately informative null or negligible effect would constitute STOP, whereas insufficient evidence should remain INCONCLUSIVE.

The central distinction is therefore among mechanistic plausibility, predictive informativeness, and decision relevance. BRAIN-TIME does not prescribe when treatment should occur; it provides a structured pathway for determining whether prospectively available recovery information identifies an intervention-specific, decision-relevant WHEN.

## Figures and Tables

**Table 1:** Conceptual separation of BRAIN-TIME from the authors’ prior frameworks [15,16].

Dimension	MAC/MAB–RCS: WHO	Reward Instability Theory: WHY	BRAIN-TIME: WHEN
**Primary question**	Who may exhibit a vulnerable regulatory profile?	Why can reward processing become dynamically unstable?	When does post-perturbation information change treatment benefit?
**Unit of analysis**	Person-level regulatory profile	Reward-system dynamics	Perturbation–trajectory–decision episode
**Central construct**	Regulatory Control State	Reward Instability	Decision-relevant recovery transition
**Defined perturbation required**	No	Not necessarily	Yes
**Sustained return required**	No	No	Yes, or explicit censoring
**Prospective landmark**	Not central	Potential vulnerability window	Mandatory and leakage-restricted
**Decisive test**	Risk stratification and personalized-intervention logic	Coherence of the dynamical account of reward dysfunction	Same-intervention treatment-effect moderation by prospective recovery position

**Table 2:** BRAIN-TIME in relation to established prediction and adaptive-intervention paradigms.

Approach	Primary Question	Typical Output	What BRAIN-TIME Adds
**Early-warning systems**	Is deterioration becoming more likely?	Risk score or warning signal [14]	Perturbation-anchored recovery construct and a treatment-effect test of temporal actionability
**Just-in-time adaptive interventions (JITAI)**	When and how should support be delivered given current tailoring information?	Context-responsive intervention rule [17]	Evidence that a prospective recovery position modifies the benefit of the same intervention
**Conventional clinical judgment or fixed elapsed time**	Has enough time passed, or does the patient appear ready?	Clinical judgment or fixed schedule	A prespecified comparator against which added recovery information must demonstrate incremental value
**Dynamic treatment regimes and SMART designs**	What sequence of treatment decisions maximizes expected outcome?	Decision rule and its estimated policy value	A prior test of whether a prospectively estimated recovery position carries treatment-effect information at all, before a sequential policy is constructed (Section Novelty Relative to Adaptive-Intervention and Causal Machine-Learning Approaches)
**Micro-randomized trials for JITAI**	What is the causal excursion effect, and how is it moderated by decision-time context?	Time-varying effect estimates and moderation by context	A perturbation-anchored, adjudicated, leakage-restricted moderator in place of an observed context feature (Section Novelty Relative to Adaptive-Intervention and Causal Machine-Learning Approaches)
**Causal machine learning for treatment-effect heterogeneity**	How does treatment effect vary across covariates?	Estimated conditional average treatment effects	Evidentiary requirements the moderator itself must satisfy before heterogeneity estimates can support a timing claim (Section Novelty Relative to Adaptive-Intervention and Causal Machine-Learning Approaches)

**Table 3:** Core BRAIN-TIME quantities and inferential roles.

Quantity	Definition	Scientific Role	What It Does Not Establish
**Observed trajectory**	Repeated observations of one prespecified domain following a defined perturbation	Describes within-episode evolution and recovery kinetics	Recovery, risk, or treatment need by itself
**Elapsed recovery time**	Time from the locked recovery origin to sustained re-entry into a prespecified individualized restoration set	Retrospective episode endpoint	What was knowable at an earlier decision landmark
**Prospective incomplete-recovery probability**	Probability, using only information available by the landmark, that recovery remains incomplete beyond a prespecified future horizon under the background policy	Decision-time estimate of prospective episode position	A biological phase, natural brain state, or treatment indication
**Recovery-position-dependent treatment effect**	Variation in the effect of the same intervention and dose across prospectively estimated recovery positions at a common locked decision landmark	Tests temporal actionability in Stage 1	An earlier-versus-later timing policy or clinical utility

**Table 4:** Minimal Prespecified Stage 1 BRAIN-TIME Proof-of-Concept Specification.

Element	Prespecified First-Study Specification
**Population**	Clinically stable outpatients with one prespecified substance use disorder receiving a stable background policy π_0_; prespecified safety exclusions.
**Perturbation**	One standardized low-intensity cue or stress challenge with a defined recovery origin and stopping rules.
**Primary recovery domain**	One domain only, for example, repeated craving ratings or a research-grade autonomic measure.
**Individualized reference**	Within-person reference estimated independently of the confirmatory episode and locked before recovery adjudication.
**Independent outward-function criterion**	A non-overlapping functional measure, such as behavioral inhibition or verified task engagement.
**Sampling**	Dense baseline characterization and fixed post-perturbation observations through confirmation of sustained re-entry.
**Decision landmark**	One fixed landmark, defined independently of the subsequent recovery trajectory.
**Prospective quantity**	Locked continuous p_i_(t,H) with prespecified no-interpretation rules.
**Randomization**	Intervention versus comparator at the common landmark; continuous p_i_(t,H) remains the primary effect modifier.
**Intervention**	One brief fixed-content intervention, held constant across recovery positions.
**Comparator**	Matched, where feasible, for time, attention, expectancy, and staff contact.
**Primary outcome**	One proximal, clinically interpretable outcome assessed in a locked window.
**Primary estimand**	A prespecified functional of Δ(p) quantifying practically meaningful treatment-effect modification.
**Current-state safeguard**	Effect modification by recovery position must persist beyond that by the current landmark value of the same domain.

The full operational specification, including GO, STOP, and INCONCLUSIVE criteria, is given in Supplementary Tables S1 and S2.

**Table 5:** Sequential BRAIN-TIME evidence gates and falsification criteria.

Gate	Question	Minimum Evidence Required	Failure Interpretation
**1.** **Measurement**	Can the prespecified trajectory be measured with sufficient reliability and temporal integrity?	Signal quality, test–retest or within-episode reliability where appropriate, timestamp integrity, preprocessing stability, and device- and context-specific validation	The candidate trajectory is not sufficiently interpretable for downstream recovery inference
**2.** **Perturbation specificity**	Is the observed trajectory attributable to the defined perturbation rather than background temporal structure?	Prespecified perturbation dose and recovery origin, biologically plausible response structure, pseudo-perturbation windows, temporal permutation or shuffled-label controls, and appropriate negative-control domains	The apparent trajectory may reflect background fluctuation, artefact, or nonspecific temporal structure
**3.** **Recovery construct**	Does sustained re-entry represent a reproducible episode endpoint distinct from transient threshold crossing?	Locked individualized restoration set, prespecified stability interval, explicit recurrence rule, and principled handling of interval/right censoring and relevant intercurrent or competing events	Elapsed time cannot be interpreted defensibly as recovery
**4.** **Prospective** **estimation**	Can incomplete recovery be estimated at the locked landmark without future-information leakage?	Development-confirmation separation, temporal information restriction, calibration, uncertainty quantification, adequate coverage, and comparison with simpler prespecified alternatives	No sufficiently reliable prospective episode-position quantity has been established
**5.** **Temporal** **actionability**	Does the effect of the same intervention vary meaningfully across prospectively estimated recovery positions?	Randomized fixed-content intervention at a common locked decision landmark, adequate treatment-arm overlap across the relevant prospective recovery-position range; prospectively specified effect-modification function or contrast with a minimum practically important criterion, evaluated with adequate precision and support	An adequately informative and sufficiently precise evaluation demonstrates a null or practically negligible treatment-by-recovery-position effect; progression to a recovery-informed timing-policy trial is not justified for the tested intervention and context
**6.** **Policy utility**	Does using recovery information to determine treatment timing improve patient-important outcomes relative to realistic alternatives?	Direct comparison of timing policies, patient-important outcomes, benefit-burden assessment, coverage, safety, fairness, and implementation feasibility	Recovery-informed timing is not justified for clinical implementation

## Data Availability

No new data were created or analyzed in this study. Data sharing is not applicable to this article.

## Supplementary Materials

The following supporting information can be downloaded at https://www.mdpi.com/article/10.3390/brainsci16101043/s1: Section S1, Table S1: Stage 1 BRAIN-TIME Prespecification Framework for Testing Temporal Actionability; Table S2: Stage-Specific GO/STOP/INCONCLUSIVE Decision Matrix; Section S2, Censoring, competing events, and intercurrent events; Section S3, Evaluation, uncertainty, and coverage requirements for the prospective recovery-position model; Section S4, Design and information requirements for the Stage 1 effect-modification test; Section S5, Construct specificity, signal-level validation, and alternative explanations of return trajectories.

## Abbreviations

BRAIN-TIME	Brain-State Reward–Control Adaptive Intervention and Neurodynamic Timing Matrix
MAC	Minimizing Addiction Craving
MAB	Modulation of Addictive Behaviors
RCS	Regulatory Control State
JITAI	just-in-time adaptive intervention
TBI	traumatic brain injury
rTMS	repetitive transcranial magnetic stimulation
GLP-1	glucagon-like peptide-1
DBS	deep brain stimulation
ICH	International Council for Harmonization of Technical Requirements for Pharmaceuticals for Human Use
TRIPOD+AI	Transparent Reporting of a multivariable prediction model for Individual Prognosis Or Diagnosis + Artificial Intelligence
PROBAST+AI	Prediction model Risk Of Bias ASsessment Tool + Artificial Intelligence
PPG	photoplethysmography
ECG	electrocardiography
HRV	heart rate variability
tACS	transcranial alternating current stimulation
EEG	electroencephalography
