# Supplementary material for: BRAIN-TIME: Testing Whether Prospective Recovery Position Modifies the Benefit of the Same Intervention in Addiction
Source: Brain Sci. 2026 Sep 29;16(10):1043. doi: 10.3390/brainsci16101043 (PMC13702700; doi:10.3390/brainsci16101043)
Supplement: Supplementary file 1 [file brainsci-16-01043-s001.zip › brainsci-4593094-supplementary.pdf]

Supplementary Materials

This Supplementary Material comprises five sections: S1, the Stage 1 prespecification framework (Sections S1.1–S1.13, with Tables S1 and S2); S2, censoring, competing events, and intercurrent events; S3, evaluation, uncertainty, and coverage requirements for the prospective recovery-position model; S4, design and information requirements for the Stage 1 effect-modification test; and S5, construct specificity, signal-level validation, and alternative explanations of return trajectories.

S1. Stage 1 BRAIN-TIME Prespecification Framework

Table S1. Stage 1 BRAIN-TIME Prespecification Framework for Testing Temporal Actionability

Table S1 operationalizes the minimal Stage 1 experiment described in Table 4 of the main manuscript. It specifies the elements that should be prospectively locked before confirmatory testing and provides an operational framework for future protocol development, ethics review, prospective registration, and statistical analysis. It is not a registered clinical-trial protocol, does not constitute ethics approval, and does not provide empirical validation of BRAIN-TIME. A concrete illustration of this specification is given in Section 10.2 of the main manuscript.

The alternatives described below represent candidate implementation choices rather than interchangeable components within a single confirmatory study. A Stage 1 implementation should prospectively select and lock one target clinical population, one perturbation class, one primary recovery domain, one decision landmark, one intervention, one comparator, and one primary outcome before confirmatory enrollment begins.

Phase / element	Prespecified element	Principal safeguard	Output
**Prerequisite development**	Primary recovery domain, acquisition and preprocessing pipeline, individualized reference, sustained-re-entry rule, censoring strategy, no-interpretation rules, and prospective recovery-position model	Analytical separation of development, held-out evaluation, and confirmatory treatment-effect testing	Locked measurement and prediction architecture
**Target population**	One prespecified substance use disorder within one defined treatment context	No uncontrolled pooling of heterogeneous disorders in the first confirmatory test	Defined target population
**Background policy**	Prespecified treatment and support environment	Explicit handling of medication changes, rescue procedures, additional treatment, relapse, and other intercurrent events	Counterfactual background-policy definition
**Eligibility**	Clinically stable participants meeting prespecified clinical and safety criteria	Independent eligibility assessment before experimental challenge and independently of the subsequent episode trajectory	Eligible participant and episode
**Individualized reference**	Within-person reference estimated independently of the confirmatory episode	Prespecified stability, adequacy, and signal-quality requirements	Locked individualized reference

**Perturbation**	One standardized, low-intensity perturbation	Fixed exposure characteristics, duration, recovery origin, termination criteria, and unconditional stop rules	Defined perturbation and recovery origin
**Primary recovery domain**	Dense repeated measurement of one prespecified domain	Timestamp integrity, acquisition-quality criteria, locked preprocessing, and no post-outcome domain switching	Post-perturbation recovery trajectory
**Recovery adjudication**	Sustained re-entry into a prespecified individualized restoration set	Restoration criterion and stability interval fixed independently of the realized episode outcome	Retrospective recovery endpoint
**Outward function**	Independent measure of preserved observable function	Conceptual and operational separation from the primary recovery trajectory	Independent validation condition
**Decision landmark**	One prespecified post-perturbation landmark	No future, post-treatment, or outcome information	Locked decision point
**Prospective recovery position**	Continuous incomplete-recovery probability	Background policy specified; model locked; temporal leakage prohibited; no-interpretation permitted	Decision-time recovery-position estimate
**Randomization**	Fixed intervention versus fixed comparator at the common landmark	Prespecified randomization unit, concealed allocation, treatment-arm overlap, and any design strata fixed in advance	Treatment assignment
**Intervention delivery**	Fixed identity, dose, duration, delivery format, and staff involvement	Fidelity and contamination monitoring	Standardized intervention exposure
**Primary outcome**	One proximal patient-important or clinically interpretable recovery-relevant outcome	Locked assessment window, sign convention, and protection against current-state confounding and mathematical coupling	Treatment response
**Confirmatory analysis**	Treatment-effect modification across continuous prospective recovery position	Prospectively specified treatment-effect-modification function or contrast, uncertainty propagation, treatment-arm overlap assessment, and minimum practically important effect-modification criterion	Stage 1 treatment-effect-modification estimate
**Decision**	GO / STOP / INCONCLUSIVE	Classification based on the cumulative evidentiary chain rather than nominal statistical significance alone	Decision regarding progression

S1.1. Inferential Scope of the Stage 1 Experiment

The Stage 1 experiment is designed to test the first causal actionability claim in BRAIN-TIME rather than clinical utility. Its central question is whether, at a common locked decision landmark, the expected effect of a fixed intervention differs across prospectively estimated positions within an unresolved post-perturbation recovery process by at least a prespecified, practically meaningful amount.

Intervention identity, dose, delivery format, comparator, decision landmark, eligibility criteria, background treatment context, and primary outcome window should therefore remain fixed across the

randomized comparison. Prospective recovery position enters the confirmatory analysis as a prespecified effect modifier rather than as a treatment-assignment rule.

A **GO Stage 1 result** would demonstrate adequately supported and practically meaningful treatment-effect heterogeneity conditional on prospectively available recovery information. It would not establish that treatment should be delivered earlier, later, or at a particular recovery-probability threshold.

A non-positive Stage 1 result may constitute either an adequately tested failure or insufficient information; the corresponding classifications are defined in Section S1.12.

S1.2. Target Quantities and Stage-Specific Estimands

BRAIN-TIME distinguishes retrospective recovery, prospective recovery position, Stage 1 treatment-effect modification, and Stage 2 timing-policy utility.

For participant i, let $t_{0,i}$ denote the locked recovery origin and the locked decision landmark. For a prespecified future horizon , the prospective incomplete-recovery probability is conceptually defined as

$$p_i(t,H) = P\left[T_i^R(\pi_0) > \left(t - t_{0,i}\right) + H \mid I_i(t)\right]$$

Here, $T_i^R(\pi_0)$ denotes the potential elapsed recovery time under the prespecified background treatment or support policy π_0, $I_i(t)$ contains only information legitimately available by the decision landmark, H and is the prespecified future horizon. The explicit use of separates the background-policy recovery quantity from the subsequent randomized Stage 1 intervention.

Let $Y_i(1)$ denote the potential primary outcome for participant or episode i under the active intervention and $Y_i(0)$ the corresponding potential outcome under the comparator. Conceptually, Stage 1 evaluates the conditional treatment-benefit function

$$\Delta(p) = E\{Y_i(1) - Y_i(0) \mid p_i(t,H) = p\}.$$

Here, $\Delta(p)$ denotes the expected benefit of the active intervention relative to the comparator at prospective recovery position p, according to the prospectively specified outcome direction and treatment-effect sign convention.

The confirmatory Stage 1 question is not whether the randomized intervention has a marginal average effect, but whether $\Delta(p)$ varies across prospectively estimable recovery positions by a prospectively specified, practically important amount. The confirmatory Stage 1 estimand should therefore be a prospectively specified functional of $\Delta(p)$ that quantifies clinically meaningful treatment-effect variation across recovery position.

The minimum practically important effect-modification criterion should be expressed on the primary outcome scale as a clinically interpretable contrast or another prospectively specified functional of $\Delta(p)$. It should not be defined solely by statistical significance of a regression interaction coefficient.

Before confirmatory registration, the Stage 1 estimand should explicitly specify the target population, treatment conditions, decision landmark, primary outcome and assessment window, intercurrent-event strategy, recovery-position effect-modification target, and population-level summary.

Where participants may contribute unequal numbers of eligible episodes, the estimand should additionally specify whether inference targets an episode-average or participant-average treatment-effect-modification quantity and how episodes will be weighted. Participants contributing more experimental episodes should not dominate the target estimand merely by virtue of greater observation frequency.

Stage 2 addresses a different causal estimand: the effect of assigning treatment according to one timing policy rather than another on a patient-important outcome.

Thus:

retrospective recovery ≠ prospective recovery position ≠ Stage 1 treatment-effect modification ≠ Stage 2 timing-policy effect.

S1.3. Target Population and Background Treatment Policy

The first confirmatory Stage 1 implementation should target one prespecified substance use disorder rather than pool heterogeneous substance use disorders unless the study is explicitly designed and sufficiently informative to evaluate disorder-specific heterogeneity.

Different substance use disorders may differ in perturbation responsiveness, treatment context, medication exposure, clinical course, and plausible recovery dynamics. Cross-disorder transportability should therefore be evaluated subsequently rather than assumed in the initial causal test.

The target population should consist of clinically stable adults with the selected clinician-confirmed substance use disorder who are receiving a prespecified stable treatment or support policy and who can provide informed consent. The operational definition and required duration of treatment stability should be specified prospectively.

The background policy π_0 must also be defined before development of the prospective recovery-position model. Its specification should include, where relevant, maintenance medication, scheduled clinical contacts, permitted supportive care, protocol-defined monitoring, and rescue procedures expected to remain available between perturbation, decision landmark, and the future recovery horizon.

Treatment discontinuation, medication change, rescue intervention, addition of therapy, relapse, unscheduled clinical escalation, or a new perturbation may change the existence or interpretation of the recovery outcome under π_0. These events should therefore be assigned prospectively to appropriate estimand-specific roles rather than automatically treated as ordinary missing observations.

Prespecified exclusion criteria should include acute intoxication or clinically significant withdrawal at the experimental session; a condition requiring urgent intervention; active suicidal ideation with intent or plan; any clinical circumstance in which experimental perturbation is independently judged to create unacceptable risk; inability to complete required supervised monitoring; and challenge-, intervention-, or measurement-specific contraindications.

Eligibility decisions must remain independent of the subsequent episode trajectory.

All initiated qualifying episodes should enter participant flow. Episodes should not be retrospectively selected because they display large displacement, prolonged recovery, visually compelling dynamics, or an apparently favorable intervention response.

Each initiated episode should instead be classified using prespecified rules as non-displacing, evaluable, interval-censored, right-censored, affected by a prespecified intercurrent or competing event, or uninterpretable. A non-displacing episode remains scientifically informative and should not be removed merely because the anticipated recovery structure failed to emerge.

S1.4. Prerequisite Development, Validation Separation, and Model Lock

Confirmatory Stage 1 testing presupposes an earlier development process that is analytically separated from confirmatory treatment-effect evaluation.

Development data may be used to define the primary recovery domain, acquisition procedures, preprocessing pipeline, signal-quality requirements, individualized reference, restoration set, sustained-re-entry stability criterion, recurrence rules, censoring strategy, no-interpretation criteria, and candidate model used to estimate $p_i(t, H)$.

These choices should be evaluated in designated development data and, where appropriate, through simulation before being locked.

Development–validation separation should reproduce the intended deployment setting rather than rely automatically on random record-level splitting. In particular, randomly assigning temporally

adjacent episodes from the same participant to development and validation sets may permit person-specific or future information to leak across stages.

Depending on the intended deployment context, evaluation may therefore use participant-level separation, forward temporal validation, site-level hold-out, or another prespecified structure that approximates prospective use.

The individualized reference should be estimated independently of the confirmatory episode. The prospective recovery-position model should likewise be developed and calibrated using preceding or otherwise held-out data compatible with the target background policy π_0 .

Before confirmatory treatment-effect testing, the following components should be version-locked: the acquisition and preprocessing pipeline; feature definitions; individualized-reference procedure; restoration criterion; model specification; calibration procedure; no-interpretation rules; software or model version; and rules used to generate $p_i(t, H)$.

Any post-lock modification should be documented as a protocol amendment and should not silently replace the prespecified primary analysis.

To make the lock verifiable rather than declarative, the locked components should be deposited before confirmatory enrollment in a repository that provides an immutable, externally time-stamped record, and the deposit identifier should be cited in the prospective registration. The deposit should include the acquisition and preprocessing specification, feature definitions, the individualized-reference procedure, the restoration criterion, the model specification and fitted parameters or model file, the calibration procedure, the no-interpretation rules, and the software environment sufficient to reproduce a prospective estimate from a permitted information set. Where data-protection constraints prevent deposit of participant-level development data, the model, code, and specification should nonetheless be deposited, and the restriction should be stated explicitly rather than used to withhold the locked artefacts themselves.

The candidate model should also be compared with simpler prespecified alternatives, including elapsed time since perturbation, current symptom or state value, recent slope, a simple temporal trend, and appropriate conventional dynamic-prediction or early-warning comparators where scientifically justified.

Increased model complexity is justified only if the recovery-position architecture demonstrates adequate prospective performance and information relevant to the inferential claim for which it is proposed.

S1.5. Episode Construction, Recovery Adjudication, and Outward Function

A confirmatory Stage 1 experiment should select one perturbation class and one primary recovery domain.

A qualifying perturbation must be temporally identifiable and characterized prospectively with respect to intensity, duration, repetition, context, prior exposure, medication status, sleep state, and relevant co-exposures where applicable.

For an initial proof of concept, a controlled low-intensity perturbation is preferred because exposure characteristics and episode origin can be constrained experimentally. Naturalistic perturbations are better reserved for subsequent transportability evaluation.

The recovery origin should ordinarily be defined as the end of the perturbation unless another biologically justified offset is specified independently of the subsequent trajectory. The recovery origin must not be moved retrospectively according to the speed, magnitude, or completeness of subsequent return.

The primary recovery trajectory should comprise one prespecified domain rather than a broad multimodal composite. Candidate implementations may include repeated craving measurements or a

research-grade physiological or behavioral measure meeting predefined acquisition, preprocessing, timing, and signal-quality requirements.

Other signals may function as covariates, negative controls, mechanistic variables, secondary outcomes, or candidate recovery domains in later studies. Their concordance with the primary domain should not be assumed.

Retrospective recovery is defined through sustained re-entry into a phase- and context-aware individualized restoration set for a prespecified stability interval. First threshold crossing alone does not constitute recovery. Transient normalization followed by rebound, renewed displacement, or unstable return remains part of the recovery trajectory.

The restoration set and stability criterion must be defined independently of the realized episode outcome. Under discrete observation, the onset of sustained re-entry may be interval-censored. Failure to demonstrate sustained recovery before the end of the observation horizon should be treated as right censoring rather than assigned an arbitrary recovery time.

Relapse, rescue treatment, treatment modification, a new perturbation, overt deterioration, participant withdrawal, or administrative study end should be assigned prospectively to estimand-specific roles before outcomes are examined. The applicable roles, classification criterion, and required sensitivity analyses are specified in Section S2.

Outward function serves an independent inferential purpose. A conceptually and operationally non-overlapping functional criterion—such as verified task engagement, behavioral inhibition, or another prespecified functional measure—should be assessed independently of the primary recovery trajectory.

Outward function must not be incorporated into or used to redefine recovery retrospectively. If outward function has already deteriorated before the locked decision landmark, the episode no longer represents the intended preserved-function condition and should be handled according to prespecified eligibility, intercurrent-event, or analysis rules.

Where restriction on preserved function influences inclusion in confirmatory analysis, the required sensitivity analyses are specified in Section S5.

S1.6. Safety and Stopping Rules

Participant safety is integral to the scientific validity of any BRAIN-TIME proof of concept.

The maximum perturbation intensity and duration should be selected conservatively on the basis of prior controlled challenge paradigms, site-specific clinical procedures, characteristics of the selected population, and institutional ethics review.

An unconditional participant-initiated and investigator-initiated stopping rule should apply throughout the challenge.

Appropriately trained clinical or research personnel should remain available during the perturbation and through a prespecified post-challenge observation period.

The protocol should prospectively specify measures used to assess craving, distress, urge to use, behavioral instability, or other relevant safety outcomes and define criteria triggering extended observation, clinical assessment, rescue procedures, or same-day follow-up.

Post-session follow-up should ascertain clinically relevant adverse events, interim substance use, unplanned treatment contact, and other events appropriate to the selected challenge paradigm.

Accumulating adverse events judged potentially related to the experimental procedure should trigger a prespecified safety review before additional enrollment continues.

No data collection should begin without approval from the responsible institutional ethics body. The present Supplementary Material defines methodological safeguards only and does not constitute ethics approval.

S1.7. Locked Decision Landmark, Prospective Recovery Position, and Coverage

The Stage 1 decision landmark must be fixed before randomized outcomes are observed and should be common across the treatment comparison.

At that landmark $p_i(t, H)$, must be generated exclusively from information contained in $I_i(t)$ —that is, information legitimately available at or before the decision point under the prespecified background policy π_0.

The prospective estimate should be time-stamped and generated before randomized treatment information can influence its value.

Post-landmark observations, completed recovery, subsequent rescue treatment, treatment modification, or outcomes resulting from randomized treatment may not modify the previously assigned recovery position.

The continuous probability $p_i(t, H)$, should remain the primary Stage 1 effect-modification quantity.

Where available information does not support a defensible estimate, the model should return a prespecified no-interpretation output; the applicable conditions, coverage reporting, and abstention analysis are specified in Section S3.

S1.8. Randomization, Repeated Episodes, and Intervention Fidelity

The unit of randomization must be specified before confirmatory enrollment.

If randomization occurs at the episode level within participants, the analysis must account for within-person dependence and possible carryover from previous randomized exposure. If treatment assignment occurs at the participant level, repeated episodes remain clustered within treatment assignment and that dependence must be incorporated into design, information planning, and analysis.

The choice of randomization unit should be determined primarily by the expected duration of intervention effects, contamination risk, and plausible carryover rather than by statistical convenience.

Where feasible, randomization may be stratified using a small number of prespecified operational strata derived from $p_i(t, H)$, to protect treatment-arm overlap across the recovery-position distribution. Such strata serve only as design devices. They must not replace the continuous $p_i(t, H)$, in the primary analysis or be interpreted as biological states.

If participants contribute repeated experimental episodes, the protocol should specify the maximum number of episodes per participant, minimum inter-episode interval, criteria for re-establishing an adequate individualized reference, and handling of medication or treatment changes occurring between episodes.

Episode order, previous challenge exposure, prior randomized treatment, and plausible carryover should be represented in the analytical design. Systematic habituation, sensitization, learning, expectancy change, or persistent intervention effects may alter subsequent perturbation responses and should not be absorbed automatically into generic within-person correlation. These processes are also relevant to the interpretation of the recovery construct itself, as set out in Section S5.

Evidence of meaningful carryover should trigger prespecified sensitivity analysis, modification of repeated-episode interpretation, or suspension of further repeated challenge where appropriate.

The intervention and comparator should be standardized with respect to identity, content, dose, duration, delivery format, device exposure where relevant, staff involvement, and permitted deviations. Intervention-fidelity and contamination procedures should be prespecified. Outcome assessors and statistical analysts should remain blinded to treatment assignment where feasible.

S1.9. Primary Outcome and Protection Against Mathematical Coupling

The initial Stage 1 experiment should select one prespecified primary outcome that is clinically interpretable and sufficiently proximal to preserve a meaningful causal relationship with the intervention.

Relapse should not ordinarily be used as the sole primary endpoint in the first controlled proof of concept because it is influenced by multiple clinical and environmental processes beyond the experimental episode.

Candidate proximal outcomes may include craving-related interference, distress burden, coping success, functional engagement, or another patient-important or recovery-relevant outcome appropriate to the selected intervention.

Whenever feasible, the primary treatment outcome should be conceptually distinguishable from the information used to estimate $p_i(t, H)$.

When the same clinical domain contributes both to $p_i(t, H)$, and to the post-intervention primary outcome—for example, when craving trajectory informs prospective recovery position and craving also contributes to treatment-response assessment—the analysis should explicitly protect against current-state confounding, regression to the mean, shared measurement structure, and mathematical coupling.

The protocol should therefore prespecify comparison with the current value of the primary domain at the decision landmark. A prespecified analysis should assess whether treatment-effect modification by prospective recovery position persists beyond treatment-effect modification by current symptom or state severity alone.

If an apparent treatment-by-recovery-position effect is fully reproduced by the current landmark value of the same domain, this would weaken or remove evidence that the recovery-position construct contributes incremental temporal actionability.

S1.10. Primary Analysis, Uncertainty, Missingness, and Information Planning

The confirmatory Stage 1 analysis evaluates variation in randomized treatment benefit across the continuous prospective recovery-position quantity $p_i(t, H)$.

The primary analysis should include randomized treatment assignment, a prespecified representation of , and the prospectively specified structure required to estimate conditional treatment-effect modification, together with covariates fixed before analysis.

Where a linear representation is scientifically justified, this may include a treatment-by-recovery-position interaction. BRAIN-TIME does not, however, require the treatment-effect function to be linear and does not assume that temporal actionability corresponds to a discrete threshold.

Where plausible non-linearity is anticipated, the confirmatory analysis may specify a constrained, low-dimensional flexible representation, such as a restricted spline or another prospectively specified smooth function. Flexible modelling should not become a retrospective search for a favorable WHEN region.

The confirmatory target remains the Stage 1 estimand defined in Section S1.2.

Statistical significance of an interaction coefficient alone is insufficient to establish temporal actionability.

Both the magnitude and uncertainty of treatment-effect modification should inform evidentiary classification. The analysis plan should therefore specify prospectively how confidence or credible intervals relative to the practically important effect criterion contribute to **GO, STOP, or INCONCLUSIVE** classification.

Because $p_i(t, H)$ is estimated rather than directly observed, uncertainty should be propagated into, or otherwise explicitly accounted for in, the Stage 1 treatment-effect-modification analysis; the sources to be addressed are enumerated in Section S3.

The uncertainty procedure should be fixed before randomized treatment-effect results are examined. Appropriate approaches may include resampling, probabilistic propagation, Bayesian posterior integration, or another validated method compatible with the final model architecture.

Missingness should not automatically be assumed non-informative. Participants experiencing greater craving, instability, adverse response, rescue treatment, disengagement, or relapse may have a different probability of missing outcome or trajectory measurements.

The primary missing-data analysis should therefore be accompanied, where appropriate, by prespecified sensitivity analyses addressing clinically plausible departures from the primary missingness assumption. Events that change the meaning of the recovery or treatment-effect estimand should not be converted automatically into conventionally imputed missing values.

A definitive sample size cannot be specified at the conceptual-framework stage. Information requirements and power should therefore be determined through preregistered simulation targeting the Stage 1 treatment-effect-modification estimand rather than the marginal intervention effect.

The simulation should incorporate, as applicable, the distribution and uncertainty of $p_i(t, H)$; episode frequency; participant-level clustering; randomization unit; treatment allocation; censoring; missingness; no-interpretation frequency; treatment-arm overlap; outcome variability; measurement error; site effects; and the minimum practically important effect-modification criterion.

S1.11. Negative Controls and Perturbation Specificity

Perturbation specificity must be demonstrated rather than assumed.

The analysis should include prespecified negative-control procedures capable of distinguishing genuine perturbation-anchored recovery from background temporal structure, baseline instability, nonspecific drift, or model artefact.

Candidate controls include matched non-perturbation windows, pseudo-perturbation windows, temporal permutation or displacement of perturbation timestamps, and verification that the apparent recovery structure is not already present immediately before the defined perturbation.

Where scientifically justified, a recovery domain with no strong mechanistic expectation of response to the selected perturbation may provide an additional specificity control.

The same trajectory-processing and recovery-adjudication procedures should be applied to negative controls wherever feasible.

If an equivalent apparent recovery pattern is reproducibly observed under temporal permutation, matched non-perturbation windows, or another appropriately designed negative-control condition, the candidate trajectory fails the perturbation-specificity requirement irrespective of its apparent predictive performance in the original data.

S1.12. GO, STOP, and INCONCLUSIVE Decision Logic

The six evidence gates defined in Table 5 of the main manuscript remain the governing evidentiary architecture.

Table S2 does not introduce additional evidence gates. It operationalizes the possible evidentiary status of each existing gate by distinguishing successful progression, adequately tested failure, and insufficient information.

A **GO** classification requires that the relevant evidence requirement is satisfied with sufficient measurement quality, support, and precision to justify progression.

A **STOP** classification requires an adequately informative evaluation demonstrating failure of the relevant evidentiary requirement.

An **INCONCLUSIVE** classification applies when available study information cannot resolve the target claim because of inadequate precision, insufficient support or treatment-arm overlap, excessive no-interpretation, incomplete negative-control evidence, protocol failure, inadequate recruitment, or another limitation that prevents an adequate test.

Failure to achieve conventional statistical significance in an inadequately informative study should not be interpreted automatically as scientific falsification.

Table S2. Stage-Specific GO / STOP / INCONCLUSIVE Decision Matrix

Evidence level	GO	STOP	INCONCLUSIVE
**1. Measurement**	Prespecified trajectory demonstrates adequate reliability, temporal integrity, and construct-appropriate measurement validity	Measurement reliability or validity is inadequate despite sufficient evaluation	Data are insufficient to assess measurement credibility
**2. Perturbation specificity**	Recovery structure is distinguishable from negative and pseudo-perturbation controls	Comparable structure is reproducibly observed in appropriate controls or before perturbation	Controls are incomplete or insufficiently informative
**3. Recovery construct**	Sustained re-entry can be reproducibly adjudicated under locked restoration and censoring rules	Restoration criterion is unstable, nonspecific, or non-reproducible despite adequate evaluation	Excessive censoring, too few evaluable episodes, or insufficient follow-up prevents resolution
**4. Prospective estimation**	$p_i(t, H)$ demonstrates adequate calibration, coverage, uncertainty characterization, support, and leakage-resistant prospective performance	Prospective performance is inadequate, or recovery position provides no defensible incremental information relative to simpler prespecified alternatives despite adequate evaluation	Calibration, support, transportability, coverage, or precision remains unresolved
**5. Stage 1 temporal actionability**	Treatment-effect modification exceeds the prespecified practically important criterion with adequate precision, support, and treatment-arm overlap	An adequately informative and sufficiently precise evaluation demonstrates a null or practically negligible treatment-by-recovery-position effect	Effect-modification estimate is too imprecise, support or overlap is inadequate, or another information limitation prevents resolution
**6. Stage 2 policy utility**	Recovery-informed timing policy improves the prespecified patient-important outcome relative to a clinically realistic alternative	An adequately tested timing policy provides no practically meaningful benefit	Policy comparison is insufficiently informative

A downstream positive result cannot compensate for either an adequately demonstrated upstream failure or an unresolved upstream evidentiary requirement.

S1.13. Progression to Stage 2 and Interpretive Boundary

Stage 1 and Stage 2 answer fundamentally different causal questions.

Stage 1 asks:

Does the benefit of the same intervention vary with prospectively estimated recovery position?

Stage 2 asks:

Does using recovery information to determine treatment timing improve patient-important outcomes relative to a prespecified, clinically realistic alternative timing policy?

A Stage 2 experiment should be undertaken only after Stage 1 produces a **GO** result demonstrating adequately supported and practically meaningful treatment-effect modification and the prerequisite measurement, perturbation-specificity, recovery-construct, and prospective-estimation requirements have also been satisfied.

A GO Stage 1 result licenses testing of a timing policy. It does not establish that the timing policy is effective.

Stage 1 need not identify a discrete probability threshold. The observed effect-modification structure may instead support a continuous, regional, or other prospectively defined timing strategy. Whatever policy is selected should be derived from prior Stage 1 evidence and locked before Stage 2 outcomes are observed.

One Stage 2 design could directly randomize participants, or—where intervention effects are sufficiently proximal and carryover can be adequately controlled—eligible episodes between a fixed-schedule policy and a prespecified recovery-informed timing policy.

Where repeated treatment opportunities occur within an episode, a micro-randomized design may instead randomize intervention availability across multiple prespecified landmarks and estimate time-varying causal effects.

The primary Stage 2 estimand should be a policy-level contrast in a prespecified patient-important outcome, not the Stage 1 conditional treatment-benefit function $\Delta(p)$ or a treatment-by-position representation of that relationship.

Only after a recovery-informed timing policy demonstrates meaningful benefit relative to a clinically realistic alternative should implementation-level evaluation proceed, including treatment burden, monitoring burden, safety, coverage, fairness, feasibility, and other patient- and system-level consequences.

S2. Censoring, Competing Events, and Intercurrent Events

Post-perturbation episodes may terminate, change meaning, or become uninterpretable for clinically different reasons. The analytical treatment of such events determines whether elapsed recovery time retains its intended biological and causal interpretation. Events should therefore be assigned prospectively to estimand-specific roles rather than absorbed into a single censoring category.

The observation structure alone already imposes two distinct forms of incompleteness. Because sustained re-entry is evaluated over a stability interval under discrete observation, its onset is localized to an interval bounded by the last observation incompatible with sustained recovery and the earliest subsequent observation sequence satisfying the prespecified stability criterion, so the recovery endpoint is interval-censored rather than observed exactly. Where sustained re-entry is not demonstrated before the prespecified observation horizon, the observed recovery time is right-censored and must not be assigned an arbitrary terminal value. These conventions are stated among the adjudication requirements in S1.5 and are not restated here.

Censoring must nevertheless be distinguished from a competing event. A competing event precludes the occurrence of the event of interest, whereas censoring denotes incomplete observation of an endpoint that could otherwise still occur. Treating a competing event as censoring implicitly asserts that the recovery endpoint remained attainable after the event, which may be biologically indefensible.

Intercurrent events constitute a third category. In the sense of the ICH E9(R1) estimand framework, intercurrent events are post-treatment events that affect either the interpretation or the existence of measurements relevant to the clinical question. Treatment discontinuation, switching or addition of therapy, and rescue treatment may therefore carry fundamentally different inferential implications from study withdrawal, administrative end of follow-up, or ordinary missingness. They should not be treated

as statistically interchangeable merely because each can interrupt an observed trajectory. Where an intercurrent event is anticipated, the corresponding estimand strategy should be prespecified rather than selected after the data are examined.

Accordingly, each event class defined in Section S1.5 should be mapped prospectively to one of the following roles:

- a competing event, where its occurrence precludes attainment of the recovery endpoint;
- an intercurrent event requiring a prespecified estimand strategy, where it alters the interpretation of the recovery endpoint without precluding it;
- administrative censoring, where observation ends for reasons unrelated to the recovery process;
- a source of missing data, where the endpoint remains defined but unobserved;
- an episode-ending condition, where the original recovery estimand is no longer interpretable and the episode should be classified according to prespecified rules rather than analyzed as a recovery time.

The same clinical event may occupy different roles under different scientific questions, so the mapping is question-specific rather than universal. Classification should be determined by whether the event changes the existence, observability, or interpretation of the target recovery endpoint, not by analytical convenience or by which category admits the simplest estimator. Where the assigned role is uncertain, the primary classification should be prespecified and accompanied by a prespecified sensitivity analysis under the alternative role.

Because BRAIN-TIME links repeatedly observed longitudinal trajectories to a time-to-recovery endpoint, joint longitudinal and time-to-event formulations provide one principled analytical family for implementation. The framework does not depend on any single model class, and the choice of estimator does not relieve the requirement to classify events before outcomes are examined. Consistent event mapping preserves the interpretation of elapsed recovery time and prevents clinically distinct post-perturbation events from being absorbed into a statistically convenient but scientifically ambiguous endpoint.

S3. Evaluation, Uncertainty, and Coverage Requirements for the Prospective Recovery-Position Model

Predictive performance for the prospective incomplete-recovery probability should be reported across all dimensions relevant to its intended decision use, rather than summarized by a single accuracy measure. The following dimensions are required.

- Calibration, assessed across the full range of predicted values and not only in aggregate, including calibration-in-the-large, calibration slope, and flexible calibration curves.
- Discrimination, reported with appropriate landmark-specific and horizon-specific measures rather than a single time-collapsed statistic.
- Overall statistical performance, combining calibration and discrimination in a single proper scoring rule.
- Classification performance, reported only where a threshold is genuinely required by the intended use, and accompanied by the justification for that threshold.
- Decision-analytic utility, evaluated over the range of trade-offs plausible for the intended decision rather than at a single operating point.

Reporting should follow the structure of the TRIPOD+AI statement, and methodological quality, risk of bias, and applicability should be appraised using PROBAST+AI. Neither reporting completeness nor a

favorable appraisal substitutes for the performance evidence itself, and neither addresses temporal actionability, which is established only by the Stage 1 treatment-effect-modification test.

The prospective incomplete-recovery probability is an estimated quantity rather than an observed biological state, and its uncertainty accumulates across several stages of its construction. The uncertainty-handling procedure should be prespecified, and the following sources should be identified explicitly and stated as either propagated or accounted for by another declared method.

- Measurement error in the constituent signals, including device-level and acquisition-level error.
- Estimation of individualized reference values used to define recovery for a given participant and domain.
- Reconstruction of the recovery trajectory from discretely sampled observations.
- Model development, including variable selection, hyperparameter selection, and any resampling used during development.
- Calibration, including any recalibration applied before model lock.
- Individual-level prediction, including finite-sample prediction uncertainty at the landmark.

Uncertainty should not be discarded by substituting a point estimate of recovery position into the Stage 1 analysis as though it were measured without error. Where full propagation is not feasible, the alternative treatment of uncertainty should be declared before the treatment-effect result is known, together with its expected direction of influence on the estimated effect modification.

A prospective model should not be required to return an apparently precise recovery-position estimate under conditions in which the estimate is not defensible. A prespecified no-interpretation output is required when signal quality falls below a declared threshold, when required inputs are missing, when the episode falls outside the region of the development data in which the model is supported, or when individual predictive uncertainty exceeds a declared limit. Each of these conditions should be operationalized before model lock.

Abstention is an outcome of the model, not an exclusion from the analysis, and must be reported as such. Coverage, defined as the proportion of eligible episodes for which an interpretable estimate was produced, should be reported overall and by prespecified subgroups, together with the reasons for abstention. Episodes receiving a no-interpretation output should be characterized and compared with interpretable episodes on baseline and episode-level features, because differential abstention changes the population to which any subsequent treatment-effect estimate applies.

Performance obtained only after excluding a large or systematically selected subset of difficult episodes does not establish a defensible prospective recovery-position quantity. Where coverage is materially below the prespecified target, the primary interpretation should address the reduced population actually analyzed, and the discrepancy should be reported as a limitation of the prospective estimation procedure rather than resolved by restricting the analysis set after the fact.

S4. Design and Information Requirements for the Stage 1 Effect-Modification Test

The Stage 1 target is variation in treatment benefit across a continuous, prospectively estimated moderator. Design and information planning must therefore address that target directly, and not be inherited from planning appropriate to a marginal treatment effect.

Support across the recovery-position distribution requires explicit attention at the design stage. Sparse observations within portions of that distribution can make apparent heterogeneity unstable, and inadequate representation of both treatment arms within a region of the distribution can prevent interpretable causal comparison in that region. The following should be prespecified.

- The region of the recovery-position distribution over which the confirmatory estimand is defined, together with the minimum support required within that region for the primary interpretation to stand.
- The minimum representation of each treatment arm required within prespecified portions of the distribution, and the design mechanism used to protect it, such as stratified randomization on operational strata.
- The planned handling of regions in which realized support falls below the prespecified minimum, including whether the primary interpretation is restricted to the adequately supported region.

Apparent effect modification driven primarily by a small number of observations at poorly supported extremes should not provide the basis for progression to a timing-policy experiment, irrespective of the nominal statistical significance of the interaction. Realized support should therefore be reported alongside the estimated treatment-benefit function, so that the regions in which the estimate is and is not interpretable are visible to the reader.

Because the moderator is estimated rather than directly observed, its uncertainty must remain visible in the Stage 1 analysis. The sources of that uncertainty are enumerated in Section S3. The procedure used to propagate it into, or otherwise account for it within, the treatment-effect-modification analysis should be prespecified before randomized treatment-effect results are examined, and should state whether the analysis treats the recovery-position estimate as measured without error, and if so, the expected direction of the resulting bias in the estimated effect modification.

Prospective recovery position should remain continuous in the primary analysis whenever feasible. Categorization is nonetheless admissible for two limited purposes, provided it is prespecified.

- Prespecified contrasts between clinically interpretable values, or between prespecified portions of the recovery-position distribution, reported as secondary quantities that aid interpretation of the continuous primary analysis.
- Limited operational strata used for design purposes only, such as stratified randomization or protection of treatment-arm overlap across the distribution.

Neither use replaces the continuous primary quantity, and neither licenses interpretation of the resulting categories as natural recovery states, latent phenotypes, biological phases, or treatment indications. Thresholds selected from the data, post hoc subgroup searches, and retrospective selection of apparently responsive regions of the distribution fall outside both permitted uses and cannot establish temporal actionability.

Evidence for progression must remain cumulative across measurement, recovery adjudication, prospective estimation, and randomized treatment-effect modification. A treatment-effect-modification analysis that reaches conventional statistical significance does not by itself warrant progression when the prospective recovery-position quantity entering it is not defensible. Progression should therefore be withheld when one or more of the following applies, even in the presence of strong discrimination.

- Calibration of the prospective recovery-position estimate is poor, or was not assessed across the range of predicted values relevant to the confirmatory estimand.
- Individual predictive uncertainty is excessive relative to the differences in recovery position over which effect modification is claimed.
- Support is inadequate in the region of the distribution driving the apparent effect modification.
- Coverage is materially below the prespecified target, or no-interpretation outputs are frequent or differentially distributed.

Where any of these conditions holds, the appropriate classification is INCONCLUSIVE with respect to the prespecified target claim rather than GO, and the deficiency should be addressed before a timing-policy experiment is considered. The corresponding stage-specific decision logic is given in Section S1.12 and Table S2.

S5. Construct Specificity, Signal-Level Validation, and Alternative Explanations of Return Trajectories

Neural, autonomic, behavioral, and subjective trajectories may evolve on different timescales within the same episode, and agreement between them cannot be assumed. Each signal class entering a BRAIN-TIME trajectory therefore requires construct-specific validation for the device, metric, population, and context in which it will be used. No wearable-derived feature may be treated as a generic surrogate for autonomic recovery, and no neural or physiological signal may be designated a brain-recovery biomarker without such validation.

For wearable autonomic measures, agreement between photoplethysmography-derived measurements and electrocardiographic references depends on the following, each of which should be fixed and reported before model lock.

- The specific device and firmware version, and the sampling and preprocessing pipeline applied to its output.
- The particular metric derived from the signal, since agreement established for one interbeat-interval metric does not transfer to others.
- Movement and motion artefact during the recording, including the physical activity context of the episode.
- The recording context more broadly, including posture, ambient temperature, and skin perfusion.

Interpretation of heart-rate variability is similarly conditional, and the following should be prespecified rather than selected during analysis.

- The signal source from which variability is derived, and whether it is electrocardiographic or photoplethysmographic.
- The recording duration, since short-term and longer-term indices are not interchangeable.
- Respiration, including whether breathing rate was controlled, recorded, or left free.
- The experimental setting, including posture and the presence of a perturbation.
- Participant characteristics, including age, medication, and comorbidity.
- The analytical procedures applied, including artefact correction, detrending, interpolation, and the software used.

Where a trajectory combines signal classes, the validation status of each constituent signal should be stated separately, and the combined trajectory should not inherit the validation of its strongest component.

A return toward an individualized reference range does not by itself establish restoration of the recovery process of interest. The following alternative explanations should be considered explicitly, and the means of addressing each should be prespecified.

- Habituation to the perturbation across repeated exposures.
- Sensitization, producing progressively larger responses to the same perturbation.
- Learning about the perturbation procedure, the measurement, or the setting.
- Compensatory strategies adopted by the participant during the episode.
- Medication effects, including changes in adherence, dose, or timing during the episode.
- Circadian phase at the time of the perturbation and across the recovery window.

- Concurrent changes in treatment or support unrelated to the studied intervention.
- Repeated perturbation exposure within a participant, including order effects across episodes.
- Drift in the measurement itself, including device, sensor, and calibration drift over the study period.
- Informative missingness, in which the probability of observation depends on the recovery process.

Each of these should be addressed through at least one declared mechanism: the perturbation design, including spacing and ordering of episodes; the measurement model, including drift correction and reference re-estimation; the covariate structure of the analysis; the prespecified intercurrent-event strategy; or a prespecified sensitivity analysis.

Where the preserved-function condition is used to restrict or stratify confirmatory analyses, the required sensitivity analyses should establish whether the Stage 1 estimand is materially altered by that restriction. At a minimum, the confirmatory analysis should be repeated without the restriction, and under at least one plausible alternative specification of the relationship between preserved function and the outcome, with any divergence between these results reported as a limitation of the causal interpretation.

If restoration cannot be distinguished from adaptation to the experimental procedure itself, the recovery construct has not been established, and the implementation should not progress beyond the recovery-construct gate irrespective of downstream predictive or causal results.

This Supplementary Material reports no empirical findings. It does not establish the existence of a prospectively identifiable decision-relevant recovery position, validate a neural or physiological biomarker, demonstrate that $p_i(t, H)$ can be estimated with clinically adequate performance, establish treatment-effect modification, or recommend a treatment-timing rule.

Its purpose is to make the BRAIN-TIME hypothesis sufficiently explicit that each inferential link can be prospectively tested, distinguished from insufficient information, and rejected when an adequately informative experiment fails to satisfy the relevant requirement.

Within this architecture, temporal prediction remains information until prospective recovery position is shown to modify treatment benefit; treatment-effect modification remains evidence rather than policy until a subsequent timing-policy experiment demonstrates that using this information improves patient-important outcomes.
